# Cardiolipin Induces CXCL9/CXCL10 Expression in Tumor-Infiltrating Lymphocytes

**DOI:** 10.3390/cells15090798

**Published:** 2026-04-28

**Authors:** Joana R. Lérias, Eric de Sousa, Carolina M. Gorgulho, Jéssica Kamiki, Patrícia A. António, Rodrigo Eduardo, Matilde Sedas, Nuno Figueiredo, Jian Han, Soon Seog Jeong, Ridong Chen, Markus J. Maeurer

**Affiliations:** 1 Immunotherapy/ImmunoSurgery Laboratory, Cell Center (Champalimaud Foundation), Avenida Brasília, 1400-038 Lisbon, Portugal; joana.lerias@research.fchampalimaud.org (J.R.L.); eric.desousa@research.fchampalimaud.org (E.d.S.); carolina.gorgulho@research.fchampalimaud.org (C.M.G.); jessica.kamiki@research.fchampalimaud.org (J.K.); patricia.antonio@research.fchampalimaud.org (P.A.A.); rodrigo.eduardo@research.fchampalimaud.org (R.E.); matilde.sedas@research.fchampalimaud.org (M.S.); 2 Hospital Lusíadas Lisboa, Rua Abílio Mendes 12, 1500-458 Lisbon, Portugal; nuno.leitao.figueiredo@lusiadas.pt; 3 iRepertoire, 800 Hudson Way, Suite 2304, Huntsville, AL 35806, USA; jhan@irepertoire.com; 4 Human Cell Co., 1701 Quincy Avenue, Suite 21, Naperville, IL 60540, USA; sjeong@humancellinc.com (S.S.J.); rchen@humancellinc.com (R.C.)

**Keywords:** tumor-infiltrating lymphocytes (TIL), pancreatic cancer, gastrointestinal cancer, mitochondria, peptide recognition, tumor-associated antigen (TAA), cytokines, T-cell receptor (TCR), mutant mitochondria, cardiolipin

## Abstract

**Background:** Cardiolipin (CL) is a phospholipid composed of a glycerol linked with two phosphatidate moieties that constitutes an integral part of the human inner mitochondrial membrane under physiological conditions. It is also vital for bacterial membrane transport and key bacterial functions associated with cell division and infection. CL is released in the cytosol or into the extracellular milieu upon cell death and during inflammation. We therefore tested the ability of CL to activate and expand tumor infiltrating lymphocytes (TIL) from patients with epithelial cancer. **Methods:** TIL were isolated from gastrointestinal tumor tissues and expanded in vitro in the presence of CL. The role of the NLRP3 inflammasome was evaluated using the specific inhibitor MCC950 and siRNA-mediated silencing of NLRP3. Phenotypic changes and T-cell potency were assessed via CXCL9/10 expression levels. To characterize the immune repertoire, deep TCR sequencing was performed to compare the TCR Vα and Vβ CDR3 regions between TIL and the corresponding tumor tissue. Recognition of autologous tumor cells and tumor-specific mutations, including mutations in KRAS and mitochondrial UQCRFS1 (D145V), was assessed using MHC class I and II restriction assays. **Results:** CL-expanded TIL exhibited increased CXCL9/10 expression, which is associated with increased potency of tissue invasion. CL-TIL exhibited broader recognition of frequently occurring KRAS mutations, and this effect could be blocked with an inhibitor (MCC950) of the NLRP3 pathway, a multiprotein inflammatory complex associated with danger signaling. TIL exhibited an enriched TCR Vα and Vβ CDR3 repertoire compared to tumor tissue, as defined by deep TCR sequencing. TCR αβ+ TIL recognized autologous tumor tissue in an MHC class I– and class II–restricted fashion, including the mutant HLA-DP–restricted mitochondrial protein associated with the electron respiratory chain complex III (UQCRFS1 D145V) presented by autologous tumor cells. **Conclusions:** CL activates the NLRP3 inflammasome pathway in TIL from patients with GI cancer and increases CXCL9/CXCL10 expression in TIL, resulting in enhanced recognition of mutant cancer–associated target epitopes, including a mitochondrial protein. CL may provide a danger signal: that facilitates TIL expansion via CL-activated pathways.

## 1. Introduction

Therapy with tumor-infiltrating lymphocytes (TIL) has been successful in patients with melanoma, achieving response rates between 42% and 70%, depending on previous treatment with checkpoint inhibitors [1]. Therapeutic success using TIL in patients with other cancers has been limited. Clinically relevant immune responses have been reported for individual patients undergoing TIL therapy, *e.g.*, patients with HPV-positive cancer [2], non–small-cell lung carcinoma (NSCLC) [3], breast cancer [4], cholangiocarcinoma [5], and colorectal cancer (CRC), where mutant KRAS G12D served as a biologically and clinically relevant target antigen [6].

We examined the expansion of TIL from patients with epithelial cancer using cardiolipin (CL), an integral component of the inner mitochondrial membrane and a constituent of bacterial pathogens. The use of CL was motivated by the observation that a distinct group of patients with cancer develop anti-phospholipid antibodies directed against 2-glycoprotein I, lupus anticoagulant, or CL, suggesting that CL exposure and subsequent immune responses occur naturally in patients with cancer. In the “Wasserman test” for syphilis, which detects antibodies against CL, false positives occurs frequently in patients with autoimmune diseases, infectious diseases, or cancer [7]. Anti-CL antibody production in patients with cancer is associated with a different phospholipid fatty acid composition in mitochondria during carcinogenesis: mitochondrial-derived CL may, in this situation, serve as an autoantigen [8]. The development of anti-CL antibodies is associated with a specific genetic background. In addition, chemotherapy leads to cell death and subsequent exposure of the inner mitochondrial membrane [9,10]. CL release as a result of cancer cell death may have different effects, e.g., a more general immunostimulatory effect on T-cells mediated via the scavenger receptor CD36, dependent on endogenous complement production in CD4+ T-cells [11], cell surface pattern recognition receptors (PRRs) including TLR2 (toll-like receptor 2) [12], NLRP3 (NOD-like receptor pyrin domain-containing protein 3), TLR9, or the N-formyl peptide receptor [13,14], which has been shown to facilitate priming of precursor T-cells [12]. We analyzed in this report the effect of CL on autologous tumor recognition by TIL and recognition of specific cancer mutations, as well as the composition of the T-cell receptor (TCR) repertoire.

## 2. Materials and Methods

CL titration. To determine the potential toxicity of CL (Echelon Biosciences, Salt Lake City, UT, USA), we performed CL titration using peripheral blood mononuclear cells (PBMCs) as indicator cells. Cells were seeded at a density of 0.5 × 10^6^ cells/well in 24-well plates, and cultures were maintained in T-Vivo medium (Lonza, Basel, Switzerland) supplemented with a cytokine cocktail of 1000 IU/mL IL-7, 150 IU/mL IL-15, and 1 IU/mL IL-21 (Bio-Techne, Minneapolis, MN, USA). On day 4, the media was transitioned to 1000 IU/mL IL-2, 1000 IU/mL IL-7 and 150 IU/mL IL-15 (Bio-Techne, Minneapolis, MN, USA) for an additional 7 days. CL was tested at 0 nM, 150 nM, 275 nM, and 450 nM at the start of the culture. Cell count and viability (via Trypan Blue exclusion) were assessed at three time points: day 0 (baseline), day 4 (post–initial expansion using IL-7, IL-15, and IL-21), and at days 11–12 (harvest, after transitioning to IL-2, IL-7, and IL-15). The concentration of 275 nM showed high cell viability and robust cell yields, comparable to vehicle-treated controls.

Effect of CL on PBMCs. To evaluate the effects of CL on cytokine production in PBMCs obtained from healthy donors, PBMCs, obtained by Ficoll density gradient of Leukopaks from the Portuguese Blood Bank, were seeded at 0.5 × 10^6^ cells/well in 24-well plates (Thermo Fisher Scientific, Waltham, MA, USA) and cultured with or without CL (275 nM CL). Cultures were maintained in T-Vivo medium supplemented with a cytokine cocktail of 1000 IU/mL IL-7, 150 IU/mL IL-15, and 1 IU/mL IL-21. On day 4, the media was transitioned to 1000 IU/mL IL-2, 1000 IU/mL IL-7, and 150 IU/mL IL-15 for an additional 7–8 days. Supernatants were collected at two distinct time points: day 4 (initial expansion phase) and days 10–11. Cytokines were quantified using a Legendplex™ Multi-Analyte Flow Assay Kit (Human Inflammation panel 1, BioLegend, San Diego, CA, USA) according to the manufacturer’s instructions. Samples were acquired on a CytoflexLX (Beckman Coulter, Brea, CA, USA), and data were analyzed using Legendplex™ Data Analysis Software (version 1 May 2025; BioLegend, San Diego, CA, USA).

Tumor microfragment culture and secretome analysis. To investigate the effect of CL on tumor tissue, surgically resected tumor microfragments (n = 3–4 specimens/individual tumor sample) were seeded in 24-well plates. Fragments were cultured for 3–4 days in the presence or absence of 275 nM CL, with or without the addition of the IL-7/IL-15/IL-21 cytokine cocktail, which was used to initiate TIL cultures. Supernatants were collected and tested for IL-1β, IL-18, and TNF-α using the Legendplex™ platform (Human Inflammation panel 1). Additionally, CXCL10 protein was quantified using a CXCL10 ELISA Kit (ab83700, Abcam, Cambridge, UK) following the manufacturer’s protocol.

TIL expansion. Tissue fragments were removed from tumor lesions, as listed in Supplementary Table S1. Individual areas of tumors were dissected in small 1–2 mm^3^ pieces and placed in 24-well plates, with two to three pieces per well, in X-vivo media (Lonza, Basel, Switzerland) supplemented with 10% Human Serum (Sigma-Aldrich, Burlington, NJ, USA) and 1000 IU/mL IL7-Fc^HC^, 150 IU/mL IL15-Fc^HC^, and 1 U/mL IL21-Fc^HC^ (Human Cell Co., Naperville, IL, USA), along with 275 nM CL at day 1, followed by a humanized anti-CD3^HC^ (30 ng/mL) (Human Cell Co., Naperville, IL, USA) and 40 Gy–irradiated allogeneic feeder cells at 10^6^ cells/well at day 2 admixed from three individual healthy donors. Feeder cells were added to TIL for expansion as needed during the expansion period, along with a humanized anti-CD3^HC^ monoclonal antibody (30 ng/mL) at a feeder cell:TIL ratio of 5:1. Tumor pieces were removed after 7 days, and the TIL medium changed to 1000 IU/mL Fc-IL2^HC^, 1000 IU/mL IL7-Fc^HC^, and 150 IU/mL IL15-Fc^HC^ between days 4 and 6 for further expansion. Cells were split as needed and expanded up to 1 × 10^9^ TIL for testing. To test for differences in a TIL potency release assays, TIL were also expanded as described above, yet with cytokines supplied by Bio-Techne, expanded first with 1000 IU/mL IL-7, 150 IU/mL IL-15, and 1 IU/mL IL-21, followed by 1000 IU/mL IL-2, 1000 IU/mL IL-7, and 150 IU/mL IL-15 after day 4. CL was used as described above during TIL expansion.

CL effect on TIL. To evaluate the effects of CL on cytokine production, TIL were cultured as described above and seeded at 0.5 × 10^6^ cells/well in 24-well plates, washed two times in T-Vivo medium (Lonza, Basel, Switzerland), and subsequently cultured with or without 275 nM CL. TIL were maintained in T-Vivo medium with a cytokine cocktail containing 1000 IU/mL IL-2, 1000 IU/mL IL-7, and 150 IU/mL IL-15. Supernatants were collected on day 3 and day 6. Cytokine concentrations were quantified using a Legendplex™ Multi-Analyte Flow Assay Kit (Human Inflammation panel 1) according to the manufacturer’s instructions. Samples were acquired on a CytoflexLX, and data were analyzed using Legendplex™ Data Analysis Software (version 1 May 2025).

NLRP3 pathway analysis. TIL were expanded from tumor tissue as described above (see TIL expansion), in the presence of CL with or without MCC950 (10 µM/mL) (Invivogen, San Diego, CA, USA), a specific inhibitor of the NLRP3 pathway, starting from culture initiation throughout the entire expansion process. TIL were tested for recognition of a panel of 26 KRAS peptides (listed in Supplementary Table S3), as described below (see Peptide recognition assay). Both CL and MCC950 were maintained throughout the 7-day incubation period testing anti-KRAS reactivity. The number of peptide targets, as well as the amount of IFN-γ in pg/mL/TIL/target, was analyzed and compared in the MCC950-positive versus -negative groups defined by IFN-γ production using an IFN-γ ELISA kit (3420-1H-6, Mabtech, Cincinnati, OH, USA), according to the manufacturer’s instructions. The initial seeding of TIL was 10^4^ cells/well, and the harvest of individual supernatants after 7 days represents the net effect of IFN-γ production resulting from T-cell proliferation and IFN-γ production/cell.

siRNA-mediated NLRP3 knockdown and functional validation. For NLRP3 gene silencing, expanded TIL were transfected with NLRP3-specific siRNA or a non-targeting scrambled control siRNA (ThermoFisher Scientific, Waltham, MA, USA). Transfections were carried out using Lipofectamine RNAiMAX (ThermoFisher Scientific, Waltham, MA, USA) according to the manufacturer’s instructions. A “nil” control (non-transfected cells) was included in all experiments to assess baseline cellular immune effector function. After transfection, TIL were cultured in T-Vivo medium supplemented with a cytokine cocktail of 1000 IU/mL IL-7, 150 IU/mL IL-15, and 1 IU/mL IL-21. TIL were incubated for 72 h at 37 °C to allow for optimal protein knockdown before being harvested for functional assays. The efficiency of the NLRP3 knockdown was validated by flow cytometry using the primary antibody anti-NLRP3 (ab263899, Abcam, Cambridge, UK), secondary antibody IgG α-Rabbit H&L-AF488 (ab150077, Abcam, Cambridge, UK), and intracellular staining with PerFix-nc kit (Beckman Coulter, Brea, CA, USA), according to the manufacturer’s instructions. Samples were acquired on a CytoflexLX (Beckman Coulter, Brea, CA, USA), and data were analyzed using the FlowJo software (version 10.10.1, BD, Franklin Lakes, NJ, USA).

Peptide recognition assay. Synthetic peptides obtained from Peptides&Elephants (Hennigsdorf, Germany), derived from shared tumor-associated antigens (TAAs) (listed in Supplementary Table S6) or derived from specific mutations (listed in Supplementary Tables S8–S11) detected by whole tumor exome sequencing, as previously described [15], were used at 1 µg/well in duplicate; anti-CD3^HC^ at 30 ng/mL or PHA (5 µg/mL) (Sigma-Aldrich, Burlington, NJ, USA) was also used. TIL were seeded at 10^4^/well in TIL medium containing 100 IU/mL of IL7-Fc^HC^ and 100 IU/mL of IL15-Fc^HC^ (Human Cell Co., Naperville, IL, USA) without serum for 6–12 h, followed by the addition of 10% human serum to the peptide–TIL mix, as described previously [16]. T-cell responses were tested using an IFN-γ ELISA (3420-1H-6, Mabtech, Cincinnati, OH, USA) according to the manufacturer’s instructions.

Blocking antibodies. MHC class I responses were blocked using the mAb clone w6/32 murine IgG2a (MA1-19027, ThermoFisher Scientific, Waltham, MA, USA) at 10 µg/mL, HLA-DR clone L243 murine IgG2a, (307602, BioLegend, San Diego, CA, USA) at 10 µg/mL, anti-HLA-DP clone B7/21 murine IgG3 (ab20897, Abcam, Cambridge, UK) at 10 µg/mL, anti-CD1d clone CD1d42 murine IgG1 (550254, BD, Franklin Lakes, NJ, USA) at 10 µg/mL, and isotype control antibody murine IgG2a (400202, BioLegend, San Diego, CA, USA) at 10 µg/mL.

Tumor recognition assay. Autologous tumor tissue, confirmed by standard histology, was immediately stored at −80 °C and subsequently thawed for T-cell recognition assays. Briefly, small pieces of autologous tumor (1–2 mm^3^) were removed from tumor areas confirmed to contain tumor cells by standard pathology. Tumor cell recognition was assessed in duplicate by measuring IFN-γ production by ELISA, as described in detail above. TIL and TCR αβ+ TIL lines were incubated with autologous tumor pieces in X-Vivo media supplemented with 10% HS in the presence of Fc-IL2^HC^ (100 IU/mL) and IL15-Fc^HC^ (100 IU/mL).

Flow cytometry. TIL were immunophenotyped using DuraClone IM T-cell Subsets (B53328, Beckman Coulter, Brea, CA, USA), and the following drop-in mAbs were added: anti-LAG3 BV650 (clone 11C3C65, BioLegend, San Diego, CA, USA) and anti-CD95 BV785 (clone DX2, BioLegend, San Diego, CA, USA). TIL were also analyzed using anti-CD4 Pacific Blue (clone 13B8.2, Beckman Coulter, Brea, CA, USA), anti-CD8 KromeOrange (clone B9.11, Beckman Coulter, Brea, CA, USA), anti-LAG3 BV650 (clone 11C3C65, BioLegend, San Diego, CA, USA), anti-CD95 BV785 (clone DX2, BioLegend, San Diego, CA, USA), anti-CD103 FITC (clone 2G5, Beckman Coulter, Brea, CA, USA), anti-CD39 ECD (clone TU66, BioLegend, San Diego, CA, USA), anti-CCR7 PE (clone G043H7, Beckman Coulter, Brea, CA, USA), anti-TCR pan-γ/δ PC5.5 (clone IMMU-510, Beckman Coulter, Brea, CA, USA), anti-CD69 PC7 (clone FN50, BioLegend, San Diego, CA, USA), anti-4-1BB (clone 4B4-1, BD, Franklin Lakes, NJ, USA), anti-CD45RA A700 (clone 2H4, Beckman Coulter, Brea, CA, USA), anti-CD3 APC-Cy7 (clone UCHT1, BioLegend, San Diego, CA, USA), and LIVE/DEAD^TM^ Fixable Yellow Dead Cell (L34959, ThermoFisher Scientific, Waltham, MA, USA). After 15 min of incubation in the dark with antibodies, cells were washed in PBS-2% FBS, and signals were acquired using a Cytoflex LX (Beckman Coulter, Brea, CA, USA). FlowJo software (BD, Franklin Lakes, NJ, USA) was used for data analysis. Gating was performed by first identifying the lymphocyte population via FSC/SSC, followed by doublet exclusion and live/dead determination. T-cell subsets were defined within the live CD3+ gate. Analysis was performed using FlowJo software (BD, Franklin Lakes, NJ, USA), and a minimum of 10,000 events were recorded for each sample to ensure statistical robustness.

CD107a assay. This assay evaluates the degranulation capacity of TIL as a surrogate marker for cytotoxic activity. During the exocytosis of lytic granules, the vesicle-associated membrane protein CD107a is transiently expressed on the cell surface. This “membrane flip” exposes an epitope detectable by anti-CD107a antibodies, providing a robust indication of effector function alongside the secretion of cytokines and other immune mediators. Briefly, positive control cells (cultured for 3 days with 100 IU Fc-IL2^HC^ and 150 IU IL15-Fc^HC^) were stimulated with 1 µg/mL of PMA (P1585-1mg, Merck, Darmstadt, Germany); non-stimulated cells served as the control, and samples were tested in triplicate. The protein transport inhibitor monensin (51-2092kz, BD, NJ, USA) and anti-CD107a PE antibody (clone H4A3, 555801, BD, Franklin Lakes, NJ, USA) were added for 2 h. Then, extracellular staining was performed using anti-CD3 PE-Cy7 (clone UCHT1, 563423, BD, Franklin Lakes, NJ, USA), anti-CD4 V450 (clone RPA-T4, 561838, BD, NJ, USA), and anti-CD8 APC-Cy7 (clone SK1, 557834, BD, Franklin Lakes, NJ, USA). We considered samples to be positive for degranulation when PMA-stimulated cells presented with at least two times the standard deviation of CD107a+ events compared to the negative (medium) control.

Regulatory T-cell (Treg) detection. To detect regulatory T-cells (Tregs), cells were first stained with anti-CD3 PE (clone UCHT-1, 555749, BD, Franklin Lakes, NJ, USA), anti-CD4 V450 (clone RPA-T4, 561838, BD, Franklin Lakes, NJ, USA), anti-CD8 APC-Cy7 (clone SK1, 557834, BD, Franklin Lakes, NJ, USA), anti-CD25 PE-Cy7 (clone 2A3, 335824, BD, NJ, USA), and anti-CD127 APC (clone R34.34, B42026, Beckman Coulter, Brea, CA, USA) for 15 min on ice and then washed and permeabilized using BioLegend’s True-Nuclear Transcription Factor Buffer Set (424401, BioLegend, San Diego, CA, USA) according to manufacturer’s instructions. Cells were then stained intracellularly with anti-FOXP3 Alexa488 antibody (clone 259D/C7, 560047, BD, Franklin Lakes, NJ, USA), anti-CD3 PE (clone UCHT-1, 555749, BD, Franklin Lakes, NJ, USA), or the IgG1 isotype control Alexa 488 (557702, BD, Franklin Lakes, NJ, USA). Treg cells were identified by flow cytometry as CD3+ CD4+ CD25high CD127-FoxP3+).

IFN-γ induction assay. This assay measures IFN-γ production after anti-CD3–induced activation during 24 h in 10^5^ cells. Briefly, TIL were seeded in triplicate (10^5^ cells/well) in Lonza X-vivo media (Lonza, Basel, Switzerland) supplemented with 10% Human Serum (Sigma-Aldrich, Burlington, NJ, USA), 1000 IU/mL IL7-Fc^HC^, 150 IU/mL IL15-Fc^HC^, and 1 IU/mL IL21-Fc^HC^ (Human Cell Co., Naperville, IL, USA) in the presence of anti-CD3 at 30 ng/mL (clone OKT3, 317302, BioLegend, San Diego, CA, USA). Negative controls were TIL cultured in medium alone without TCR crosslinking. After 24 h of incubation at 37 °C and 5% CO_2_, supernatants were harvested, and an IFN-γ ELISA (3420-1H-6, Mabtech, Cincinnati, OH, USA) was performed according to the manufacturer’s instructions.

RNA extraction and sequencing. RNA from TIL was isolated using the RNeasy Mini Kit (Qiagen, Venlo, The Netherlands). Sequencing library preparation was performed using the KAPA RNA HyperPrep Kit with KAPA RiboErase Kit (HMR) (Roche, Basel, Switzerland). Sequencing was carried out on a NovaSeq 6000 system with 100 bp paired-end reads (Illumina Inc., San Diego, CA, USA). Adapters were removed using Skewer [17]. STAR was used to map reads to the human genome, version GRCh38, obtained from Ensembl release 104 [18] and to count the number of reads per gene [19]. For gene signature analysis, mRNA transcripts were normalized using transcripts per million (TPM), and heatmaps were built to analyze the TIL and to evaluate the Th1, Th2, Th17, Tfh, and Treg profiles.

Differential gene expression analysis. Genes with extremely low counts across samples were removed. DESeq2 was used with a false discovery rate < 0.10 and with a log_2_(fold change) > 0.6 or <−0.6 as cut-off values to estimate differential gene expression between TIL grown with and without CL [20].

DNA extraction and sequencing. DNA from FFPE (formalin-fixed, paraffin-embedded) tumor tissue was isolated using an Allprep DNA/RNA FFPE Kit (Qiagen, Venlo, The Netherlands), and DNA from blood was isolated using the QIAsymphony DSP DNA Mini Kit (Qiagen, Venlo, The Netherlands). Sequencing library preparation was performed using the Twist Human Core Exome Plus Kit (Twist Bioscience, San Francisco, CA, USA). Sequencing was carried out on a NovaSeq 6000 system with 100 bp paired-end reads (Illumina Inc., San Diego, CA, USA). Adapters were removed using Skewer [17]. Exome sequencing data were aligned using the Burrows-Wheeler Aligner [21] to the human genome, version hg19. Duplicates were marked using Picard’s [22] MarkDuplicates tool, and base recalibration was performed using GATK [23].

TCR sequencing and analysis. NGS libraries covering human the TRA and TRB CDR3 regions were prepared using iR-RepSeq+ dam-PCR technology (iRepertoire Inc., Huntsville, AL, USA). After quality control, the sequencing libraries were pooled, and sequencing was carried out on a NextSeq 550 system with 300 base pair (bp) single-end reads (Illumina Inc., San Diego, CA, USA). The raw data were analyzed using iRepertoire’s proprietary software (version YPL6) pipeline to remove sequencing artifacts and further mapping to reference sequences to identify CDR3 regions. Individual CDR3 regions were visualized according to their frequencies in the total reads of the TCR chains and color-coded based on their association with TCR variable chain families using TCRcloud [24]. For analysis of peptide-reactive TIL, any CDR3 detected in the control sample (medium or positive control) was removed.

MHC typing. Typing for patients D1309 and D1313 was performed with the Fluogene HLA Typing system (Inno-Train, Taunus, Germany), and typing for patients D1688 and D3731 was performed using the One Lambda AllType FASTplex NGS 11 Loci Kit (ThermoFisher Scientific, Waltham, MA, USA). Sequencing was performed with the Ion Torrent system (ThermoFisher Scientific, Waltham, MA, USA).

Spatial transcriptomics. Spatial transcriptomics libraries were prepared using the Visium Spatial Gene Expression Technology for FFPE tissue (10× Genomics, Pleasanton, CA, USA) according to the manufacturer’s instructions. Sequencing was carried out on a NovaSeq 6000 system with 28, 10, 10, and 90 bp asymmetric paired-end reads. The data processing was performed using Space Ranger software (10× Genomics, Pleasanton, CA, USA), and further visualization and analysis were performed in the Loupe browser (10× Genomics, Pleasanton, CA, USA).

Statistical analysis. To evaluate whether there was a significant difference between the means of two groups, we performed a Student’s *t*-test using GraphPad Prism version number 8.0.2. A difference was considered to be statistically significant when the *p* value was lower than 0.05.

## 3. Results

**Figure 1:**
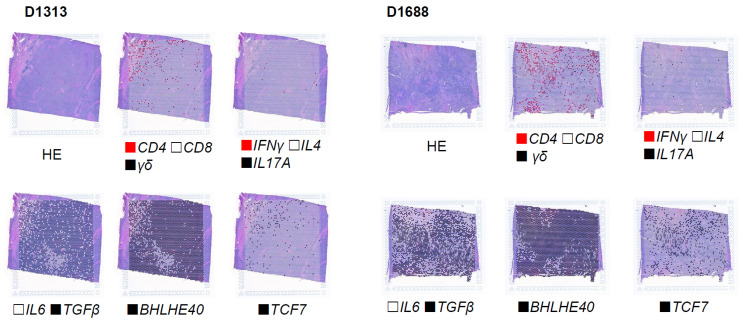
T-cells infiltrate cancer tissue. Spatial transcriptomics analysis of a neuroendocrine pancreatic tumor (D1313) and an intrahepatic cholangiocarcinoma (D1688) showing HE staining followed by the superimposition of spots in different colors representing the presence of transcripts for *CD4* (red), *CD8* (white), *γδ* (black), *IFNγ* (red), IL4 (white), *IL17A* (black), *IL6* (white), *TGFβ* (black), *BHLHE40* (black), *TCF7* (black), *γδ* and *CD1d* (black), and *γδ* and *BHLHE40* (black).

**Figure 2:**
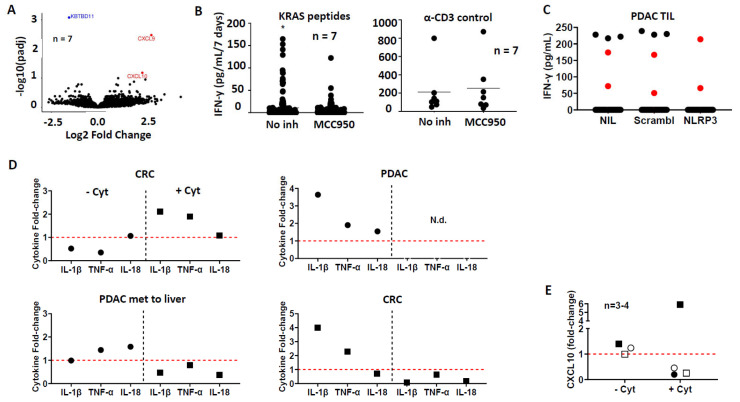
Cardiolipin-mediated NLRP3 signaling orchestrates a pro-inflammatory tumor microenvironment and facilitates TIL reactivity. (**A**) RNA sequencing analysis of TIL expanded with 275 nM CL compared to vehicle controls. Differential expression analysis identified upregulation of the CXCR3 ligands CXCL9 and CXCL10, alongside downregulation of KBTBD11. (**B**) To assess the role of the NLRP3 inflammasome in antigen recognition, TIL were expanded in the presence or absence of the specific inhibitor MCC950 (10 µM). Functional reactivity was evaluated against a library of KRAS wild-type (WT) and mutant peptides. A significant reduction in the breadth of KRAS mutation recognition (measured by IFN-γ) was observed in the MCC950-treated group (* *p* < 0.01). Stimulation with α-CD3 (right panel) confirmed comparable T-cell fitness and viability between both groups (mean 253 pg/mL vs. 211 pg/mL; *p* > 0.05), ensuring that the observed inhibition was not due to non-specific toxicity. (**C**) To validate the pharmacological findings, TIL were transfected with either a non-targeting (scrambled) siRNA or an NLRP3-specific siRNA for 72 h. Following knockdown, TIL were co-cultured with KRAS mutant peptides for 72 h to assess functional recognition. Reduction in IFN-γ production was noted in the NLRP3-silenced group as compared to both the scrambled control cells and the untreated (nil) cells; red: positive controls (PHA and anti-CD3 crosslinking). (**D**) Tumor microfragments were cultured with or without 275 nM CL, in the presence or absence of the cytokine cocktail (IL-7/IL-15/IL-21). Analysis of the cell culture supernatants showed that CL induces a robust increase in the pro-inflammatory cytokines IL-1β, IL-18, and TNF-α (measured by Legendplex). Notably, this induction occurred independently of exogenous cytokine supplementation. (**E**) Consistent with the transcriptomic data in (**A**), CL-treated microfragments showed an increase in secreted CXCL10, as measured by ELISA. Statistical significance was determined using a two-tailed Student’s *t*-test for head-to-head comparisons. A *p*-value < 0.05 was considered statistically significant.

TIL from patients with epithelial cancer (tumor histology shown in Supplementary Table S1) were expanded from tumor tissue resected from patients with gastrointestinal cancer. Spatial transcriptomics analysis of the tumor microenvironment revealed minimal *IFNγ*, *IL4*, or *IL17* signals, yet high *IL6* and *TGFβ* mRNA levels. We also observed high expression levels of *Bhlhe40* and *TCF7* (Figure 1), genes associated with tissue-resident T-cells and T-cell polyfunctionality [25]. TIL from tumor fragments were expanded in the presence of IL7-Fc^HC^ (1000 IU/mL), IL15-Fc^HC^ (150 IU/mL), and IL21-Fc^HC^ (1 U/mL), with or without CL (275 nM). To ensure that the immunomodulatory effects were not confounded by toxicity, we performed a dose titration of CL ranging from 0 nM to 450 nM in PBMCs from healthy donors. A CL concentration of 275 nM demonstrated no adverse effects on cell viability or total cell number (Supplementary Figure S1A,B). In addition, CL was diluted in chloroform:methanol for a stock solution of 1 mg/mL, and the amount vehicle present at the final concentration was negligible. Gene expression analyzed by bulk RNA sequencing revealed that CL did not lead to statistically significant differences in T-cell subpopulations based on frequency of CD45RA and CCR7 expression, nor expression of T-cell activation or exhaustion markers (Supplementary Figure S2A–D). No differences in IFN-γ production upon T-cell receptor (TCR) crosslinking with anti-CD3 and no differences in the frequency of Tregs were observed in CL- versus CL+ TIL cultures (Supplementary Figure S3A,C). CD107a expression upon PMA activation was identical in 2/3 TIL (Supplementary Figure S3B). Expansion of TIL using non–IgG-fused cytokines showed similar values regarding TIL release criteria established in our laboratory, i.e., cellular phenotype, PMA-induced CD107a expression, IFN-γ production upon TCR crosslinking with anti-CD3, and low Treg frequency (Supplementary Figure S3D–G). Next, we examined whether the addition of CL would affect signature cytokines associated with Th1/Th2/Th17 T-cell populations. Gene signatures exhibited immune gene expression profiles associated with T-cell memory, effector differentiation, or T-cell exhaustion, yet no statistical differences were found in gene signatures between CL-positive and CL-negative expanded TIL cultures (Supplementary Figure S4). CL-expanded TIL and four out of seven PBMC samples exhibited increased production of IL-1β, TNF-α, or IL-18 as compared to CL-negative controls (Supplementary Figure S1C,D; Supplementary Table S2). To further evaluate the impact of CL on the inflammatory milieu, we treated tumor microfragments with or without CL in the presence or absence of cytokines. We observed that CL increased the production of CXCL10, IL-1β, TNF-α, and IL-18, mainly in cells treated with CL but without cytokines (Figure 2D,E). Consistent with increased CXCL10 protein production, mRNA expression of *CXCL9* and *CXCL10* was found to be increased in CL-expanded TIL, while expression of KBTBD11 was found to be reduced (Figure 2A). Collectively, these data suggest that, while CL does not alter the phenotype of T-cell subsets, it modulates the pro-inflammatory cytokine profile within the tumor microenvironment, providing a “danger signal” characterized by CXLC10, IL-1β, TNF-α, and IL-18.

CL may affect cells on different levels, with one of them being activation of the NLRP3 pathway. To investigate whether the NLRP3 pathway represents a biologically relevant pathway for CL, we expanded TIL with CL in the absence or presence of the NLRP3 inhibitor MCC950. Subsequently, we assessed TIL functionality by measuring IFN-γ production against a panel of wildtype and mutant KRAS epitopes (Figure 2B, Supplementary Table S3). TIL expanded in the presence of MCC950 exhibited a reduction in both the breadth of KRAS epitope recognition and the magnitude of IFN-γ production compared to CL-expanded TIL alone. Specifically, the number of recognized epitopes decreased from 50 (without inhibition) to 42 (with NLRP3 inhibition), suggesting that CL promotes the expansion of T-cells directed against molecularly defined epitopes via NLRP3 signaling (Figure 2B). This effect was recapitulated in TIL isolated from a patient with PDAC using siRNA-mediated knockdown of NLRP3, with a 70% reduction in gene expression (25.8% NLRP3 expression in the scrambled siRNA group compared to 7.7% in NLRP3 siRNA–transfected cells). Consistent with the inhibitor data, control TIL (nil) or TIL transfected with the scrambled control both recognized 3 / 25 KRAS peptides, namely KRAS_G12S_, KRAS_G13A_, and KRAS_WT_. In contrast, NLRP3 silencing resulted in recognition of 0/25 KRAS peptides, as defined by IFN-γ production, while IFN-γ production using maximal T-cell stimulation (positive control, α-CD3, and PHA) remained intact (Figure 2C).

**Figure 3:**
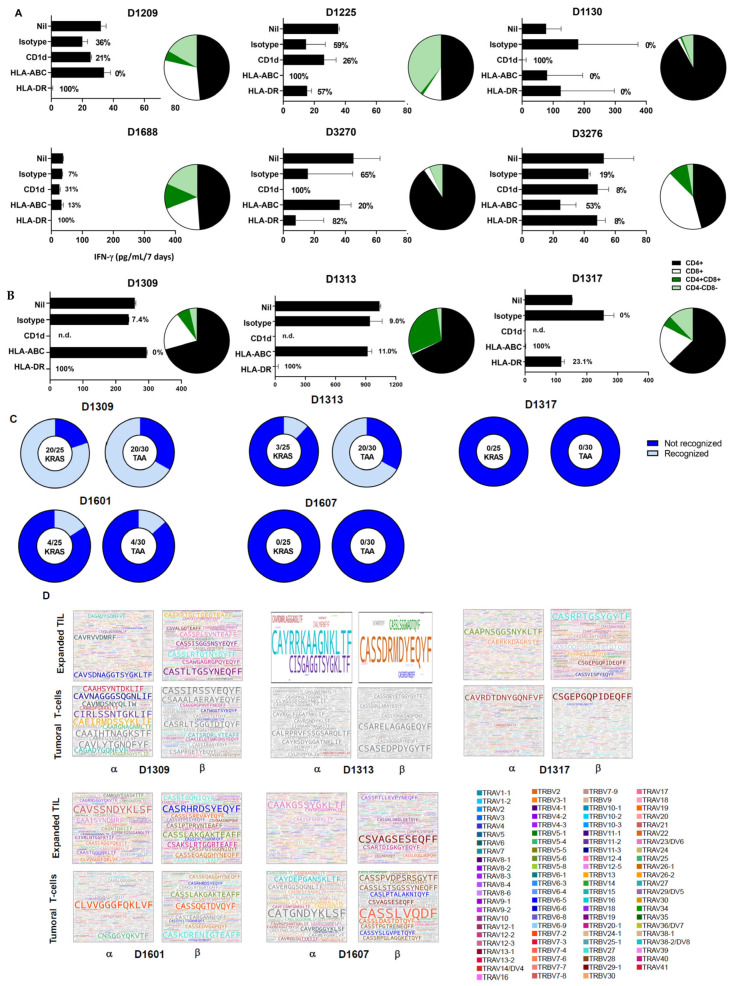
Broadened TCR diversity and multi–HLA-restricted autologous tumor recognition in TIL following CL-supplemented expansion. (**A**) TCR αβ+ T-cells from six different TIL (phenotype defined in pie charts, based on CD4+, CD8+, DN CD4-CD8- double-negative, or DP CD4 + CD8+ T-cell populations) were incubated with autologous tumor pieces and tested for tumor recognition, as measured by IFN-γ production, without the use of any blocking antibody (nil) or using an antibody isotype (isotype IgG1), a CD1d-blocking antibody (clone CD1d42), an HLA-ABC–blocking antibody (clone w6/32), or an HLA-DR–blocking antibody (clone L243). The percentages refer to the reduction in IFN-γ production as compared to the “nil” group. Autologous tumor material recognition could be blocked with the anti-HLA-DR–restricted mAb in TIL from patients D1209, D1688, and D3270. Preferential blocking with the anti-MHC class I mAb was detected in TIL from D3276, and the CD1d mAb blocked tumor recognition in TIL from patient D1130. (**B**) Recognition of autologous tumor cells by unsorted TIL expanded from three different tumors (D1309 pancreatic adenocarcinoma, D1313 neuroendocrine pancreatic, D1317 pancreatic adenocarcinoma), with the corresponding T-cell phenotype frequency presented as pie charts. TIL were incubated with autologous tumor pieces and tested for tumor recognition, as measured by IFN-y production without any blocking antibody (nil) or using an isotype control antibody (isotype IgG1), a CD1d-blocking antibody (clone CD1d42), an HLA-ABC–blocking antibody (clone w6/32), or an HLA-DR–blocking antibody (clone L243). The percentages refer to the reduction in IFN-y production relatively to the “nil” group; D1309 and D1313 TIL recognized autologous tumor in an HLA-DR–restricted fashion. Tumor recognition by TIL from D1317 was ablated using the anti-MHC class I–restricted mAb w6/32, and the anti-HLA-DR mAb decreased IFN-γ production to about 23%. (**C**) Recognition of KRAS peptides or tumor-associated antigens (TAA) by TIL. (**D**) CDR3 sequences from the TCR α-chain or β-chain sequences in matching tumor samples and in TIL. TCR CDR3 transcripts shared between tumor tissue and TIL are colored. The color code represents the variable gene of the TCR. Grey: TCR transcripts are not shared between TIL and tumor tissue.

Next, we tested TIL reactivity directed against autologous tumor cells using tumor mini-fragments as targets by adding blocking antibodies against MHC class I or HLA-DR, an antibody against CD1d, or an isotype control antibody. With regard to blocking the MHC class I–TCR interaction, 2/9 TIL cultures exhibited a strong (80–100%) reduction in IFN-γ production, 1/9 TIL cultures exhibited 30% to 60% inhibition, and 5/9 TIL cultures exhibited less than 30% inhibition. Concerning blocking with an anti-HLA-DR antibody, 5/9 TIL cultures exhibited a strong (80–100%) reduction in IFN-y production, 1/9 TIL cultures exhibited 30% to 60% inhibition, and 3/9 TIL cultures exhibited less than 30% inhibition (Figure 3A,B).

Sequencing of the TRA and TRB CDR3 regions in TIL and the corresponding tumor tissue revealed a broad TCR repertoire, listed in Supplementary Table S4, with TIL from patient D1309 showing the lowest number of individual TCR transcripts (2392 TCR α-chain and 2465 TCR β-chain transcripts) and TIL from patient D1317 exhibiting the highest number (31,666 α-chain and 40,027 TCR distinct β-chain transcripts). All TIL samples exhibited a higher number of (detectable) distinct TCR Vα CDR3 motifs as compared to the matching tumor tissue, and 3/5 TIL samples exhibited a higher number of (detectable) distinct TCR Vβ CDR3 motifs as compared to TCR transcripts in matching tumor tissue (Figure 3D and Supplementary Table S4).

**Figure 4:**
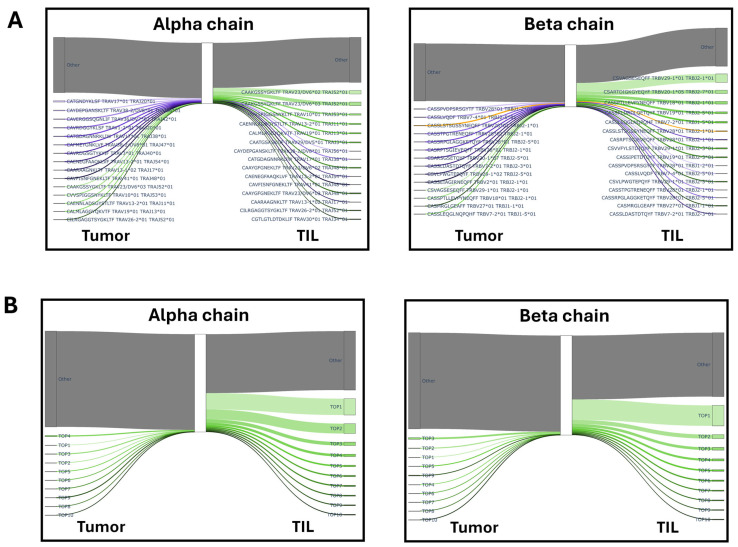
Clonal enrichment of TCR transcripts in TIL compared to tumor tissue. (**A**) Sankey plot illustrating the top 10 most frequent TCR CDR3 sequences in tumor tissue and in TIL from patient D1607 as a paradigm for each TCR chain. Each line represents a unique CDR3 sequence, and the line width is proportional to the percentage of the CDR3 sequence transcript. The top 10 CDR3 sequences in tumor issue (purple) and in TIL (green) are shown; orange designates common CDR3 sequences present in TCR transcripts in tumor tissue and TIL. (**B**) Sankey plot illustrating the average of the 10 most abundant TCR CDR3 sequences in tumor tissue and corresponding TIL from five patients (D1309, D1313, D1317, D1601, and D1607). Identical color code as in (**A**).

We found clonal TCR αβ expansion in TIL as compared to the matching tissue sample. The frequencies of the top 10 TCR CDR3 clonotypes was found to be increased in 4/5 TIL samples for the TCR α-chain and in 3/5 TIL samples for the TCR β-chain (average of the frequency of top 10 clonotypes: 11.61% TCR α-chain and 12.21% TCR β-chain in tumor tissue versus top 10 distinct TCR clonotypes constituting 39.86% TCR α-chain and 35.90% TCR β-chain transcripts in TIL) (Figure 4B and Supplementary Table S5). This was statistically significant, with a *p*-value < 0.0001 for the TCR α-chain and a *p*-value < 0.05 for the TCR β-chain.

The top 10 TCR transcripts were different in tumor tissue as compared to (matching) TIL, with some exceptions, as shown in Figure 4A for patient D1607 (i.e., the CASSLSTSGSSYNEQFF CDR3 sequence (TRBV28*01)), suggesting that the TIL expansion process described in this report favored the expansion of T-cell clones with a low frequency in the matching tumor tissue. The Sankey plot, where we combined the TCR Vα and Vβ CDR3 transcripts in the TCR transcriptome analysis from matching tumor tissue and TIL from five patients, showed that the most frequent TCR CDR3 sequences in tumor tissue and TIL were not shared (Figure 4B).

**Figure 5:**
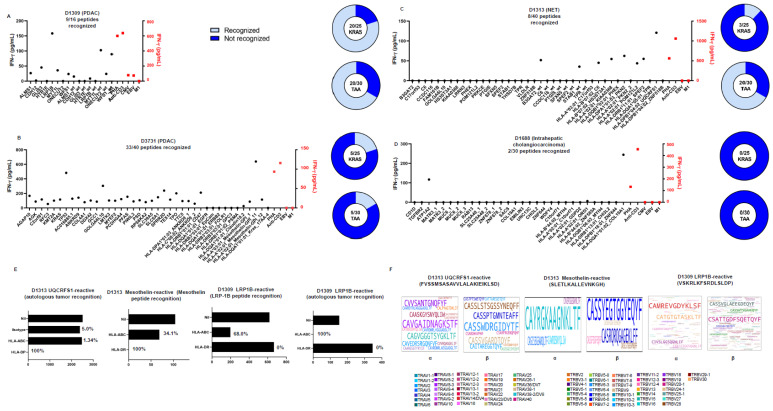
TIL recognized commonly shared tumor-associated targets and peptides representing neoepitopes, including an HLA-DP–restricted mitochondrial protein. (**A**–**D**) TIL expanded ex vivo from four different tumor types ((**A**) D1309 PDAC, (**B**) D3731 PDAC, (**C**) D1313 neuroendocrine tumor (NET), (**D**) D1688 intrahepatic cholangiosarcoma) were incubated with peptides corresponding to synthetic neoepitopes obtained by DNA exome sequencing of the autologous tumor tissue (bar graphs, specific reactivity to peptides in black and to positive controls, e.g., PHA, anti-CD3, EBV (EBNA3) or Flu-M1 in red) or with commonly shared tumor-associated antigens (TAA) (pie charts) and tested for reactivity, as measured by IFN-y production. Although at a different frequency, 4/4 TIL samples recognized neoepitopes. (**E**) Peptide-expanded TIL (UQCRFS1 and mesothelin for D1313 and LRP1B for D1309) were tested for recognition of the stimulating peptide and the autologous tumor, as measured by IFN-γ production; blocking antibodies against HLA-ABC, HLA-DP, and HLA-DR were used to gauge MHC restriction. From left to right: the UQCRFS1 peptide–expanded TIL line recognized the tumor in an HLA-DP–restricted fashion; the mesothelin peptide–expanded TIL showed 100% inhibition, as defined by IFN-γ production, using the anti-DR mAb, and 34.1% inhibition using the pan-MHC mAb, suggesting HLA-DR–restricted recognition of the autologous tumor. LRP1B peptide–expanded TIL reactivity could be decreased to 68% using the anti-MHC class I–directed mAb w6/32, and recognition of the autologous tumor tissue was blocked 100% with the anti-MHC class I reagent, but not with the anti-DR–specific mAb. (**F**) CDR3 sequences derived from the TCR α-chain or β-chain from peptide-expanded TIL (UQCRFS1 and mesothelin for D1313 and LRP1B for D1309). Colors represent the variable gene of the TCR.

TIL were then tested against peptides corresponding to a panel of commonly or frequently mutated tumor-associated antigens (Supplementary Table S6), as measured by IFN-γ production (Figure 3C). TIL recognized a broad number of KRAS mutations, as well as commonly shared tumor-associated antigens, including mesothelin or MUC4 (Supplementary Table S7). Since these target peptides are 15 mers and not tailored to the patient MHC class I or class II haplotypes, we performed DNA exome sequencing of tumor tissue from four patients, along with MHC four-digit typing, and tested TIL against a molecularly defined set of synthetic peptides representing neoepitopes. All of the TIL samples recognized the molecularly defined mutations, which are listed in Supplementary Tables S8–S11 and shown in Figure 5A–D. TIL from D1309 (PDAC) recognized 9/16 epitopes, TIL from D1313 (neuroendocrine tumor pancreatic cancer) recognized 8/40 epitopes, TIL from D3731 (PDAC) recognized 33/40 epitopes, and TIL from D1688 (intrahepatic cholangiocarcinoma) recognized 2/30 target epitopes, eliciting IFN-γ production (Figure 5A–D).

Three target epitopes were then selected for further TIL expansion to generate oligoclonal and peptide-reactive TIL lines. TCR sequencing was subsequently performed in peptide-expanded TIL, i.e., from two target epitopes (UQCRFS1 D145V, a mitochondrial-associated protein and a peptide target derived from the non-mutant tumor-associated antigen mesothelin) recognized by D1313 TIL and one target epitope recognized by D1309 TIL (LRP1B P3132S) (Figure 5A,C).

Peptide-expanded TIL were tested for recognition of the stimulating peptide and the autologous tumor, as measured by IFN-γ production; MHC restriction was tested using monoclonal antibodies directed against MHC class I (HLA-A, B, C) (clone w6/32) and against HLA-DR (clone L243), showing that recognition of the peptide HLA-DPB1*04:02_UQCRFS1 (mutant epitope from the mitochondrial protein UQCRFS1) was blocked using an HLA-DP–specific antibody (Figure 5E).

Sequencing of the human Vα and Vβ CDR3 regions of UQCRFS1-reactive TIL suggested that TCRs mediating peptide-specific recognition were oligoclonal, with the most frequent sequence being TCR CAVGAIDNAGKSTF (TRAV8-3 16.67%) (Figure 5F and Supplementary Table S12), which constituted 0.04% of TCR α-chain CDR3 transcripts in tumor tissue (Figure 5F). The most frequent TCR Vβ CDR3 sequence was CASSWDRGIDYTF (TRBV6-3 15.00%) in UQCRFS1 peptide–expanded TIL, which was below the detection limit in the matching tumor tissue (Figure 5F and Supplementary Table S12). Sequencing of the human TRA and TRB CDR3 regions of mesothelin-reactive TIL exhibited the TCR α-chain CDR3 motif CAYRGKAAGNKLTF (TRAV38-2/DV8 78.57%), which constituted 0.09% of TCR α-chain CDR3 transcripts in tumor tissue (Figure 5F and Supplementary Table S13). The top TCR β CDR3 sequence in the mesothelin-epitope specific TIL line was CASSYEGTGGYEQYF (TRBV6-5 33.33%) (Figure 5F and Supplementary Table S13). Both TCRs were below the detection limit in the matching tumor tissue, suggesting that some MHC peptide–reactive TCRs are already expanded in the tumor and can be maintained or expanded during the TIL expansion process.

Sequencing of the TRA and TRB CDR3 regions of LRP1B-reactive TIL showed CATGTGTASKLTF (TRAV17*01 13.86%), which was found to constitute 4.51% of TCR α-chain CDR3 transcripts in tumor tissue (Figure 5F and Supplementary Table S14). Of note, one of the top CDR3 TCR α-chain sequences (CAMREVGDYKLSF) in LRP1B peptide–specific TIL was derived from the same VDJ combination, but from two different alleles of the TRAV14/DV4 gene (Supplementary Table S14). The most frequent TCR β CDR3 sequence, CASSRTGIDSNQPQHF (TRBV6-1 14.44% in peptide-reactive TIL), constituted 6.90% of TCR β transcripts in tumor tissue (Figure 5F and Supplementary Table S14).

## 4. Discussion

We show here that TILfrom patients with epithelial malignancies can be readily expanded with CL to more than 1 × 10^9^ TIL. Successful expansion from PDAC-TIL has been described by us earlier [26], yet this has been performed with a different expansion protocol. In the current report, cardiolipin was used during the entire TIL expansion period. CL is an integral part of the inner mitochondrial membrane and constitutes about 20% of mitochondrial weight [27]. Autoantibodies directed against CL are detected in patients with autoimmune diseases or cancer [12], suggesting that at least humoral immune recognition takes place. The current report shows that CL facilitates expansion of T-cells directed against autologous tumor cells. CL has been described to activate, in a preclinical model, the TLR2 pathway and to bind to CD14, which facilitated activation of antigen-specific CD4+ and CD8+ T-cells recruited from the precursor T-cell pool. TLR2 engagement with and CD14-mediated direct activation of CD4+ T-cells may provide a useful danger signal to preferentially expand anti-cancer directed T-cells [12]. Of note, CL did not significantly change the T-cell phenotype or gene expression associated with the Th1/Th2/Th17 T-cell subsets, exhaustion, or T-cell activation markers, yet CL increased CXCL9 and CXCL10 gene expression (Supplementary Figures S2A and S4). Both chemokines are associated with increased invasion of T-cells into tissue, and increased CXCL9/10 serum concentrations in patients with pancreatic cancer are associated with increased survival [28]. CXCL10 is also used as a transgene in cell therapy to allow for increased tissue migration and survival of immune effector cells [29,30,31].

Beyond the expansion of T-cell clones, our study highlights the role of CL in orchestrating a pro-inflammatory environment. By treating tumor microfragments with CL, we observed a consistent increase in the production of CXCL10, IL-1β, IL-18, and TNF-α. This occurred independently of exogenous cytokines, suggesting that CL acts as a potent stimulus within the tumor architecture.

While we did not perform standard chemotaxis assays, the simultaneous upregulation of CXCL9 and CXCL10 is of high functional relevance. These CXCR3 ligands are essential for the recruitment and positioning of effector T-cells within the tumor. Furthermore, the increased production of IL-1β and IL-18 is consistent with activation of the NLRP3 inflammasome pathway. We propose that this CL-induced inflammatory “prime” lowers the activation threshold for T-cells, thereby facilitating broader recognition of KRAS mutant epitopes observed in the TIL reported here.

CL has been estimated to be released from necrotic cells, based on the observation that a single cell contains about 2.2 ± 0.6 fmol, *i.e.*, 3 pg CL/cell, which equals to 3 μg of CL released from 10^6^ cells [12]. The CL concentration used in this report of 275 nM (considering 1 mL) with a total weight of 0.37 μg (1 mL medium) equals the CL amount released by 10^6^ cells. Most TIL expansion protocols require allogeneic 30 to 55 Gy–irradiated feeder cells in the range of 400:1 or 200:1 (feeder cells:TIL) [26,32,33]. The 40 Gy–irradiated feeder cells in the current protocol were used at an unusually low ratio of 5:1 (feeder cells:TIL), and it could very well be that the addition of CL during TIL expansion may substitute for CL released from dying feeder cells in the form of “mitochondrial debris” associated with immune stimulation via PRRs, i.e., NLRP3, TLR9, TLR2, or CD14 [34]. This was supported by the observation that blocking the NLRP3 pathway via MCC950 reduced the number of different KRAS epitopes recognized by TIL (Figure 2B). CL activates the NLRP3 inflammasome, a protein complex assembled in response to pathogen-derived or host-derived stress signals leading to cytokine production [35]. The NLRP3 inflammasome activates caspase-1 in response to cellular damage, followed by production of the pro-inflammatory cytokines IL-1β and IL-18 [36,37]. The NLRP3 inflammasome has been described to assemble in CD4+ T-cells without the need for antigen presenting cells (APCs), starting a caspase-1–dependent IL-1β inflammatory signal, subsequent IFN-γ production, and Th1 differentiation in an autocrine fashion, followed by pro-inflammatory cytokine production, reminiscent of an early publication by Schwartzentruber and Rosenberg, in which TNF-α was identified as a surrogate marker of active feeder cell function for TIL expansion [38].

Of note, we found TCR αβ diversity to be increased in TIL upon expansion with the protocol described here, which has not been reported to be the case for TIL from patients with PDAC expanded with IL-2 [39]. The diversity of TCR αβ CDR3 sequences was increased, and the transcripts were distinct from the TCR transcripts found in the baseline tumor tissue (Figure 4 and Supplementary Table S4). Most of the TIL αβ TCR clonotypes were either very low in frequency or below the detection limit in tumor tissue. This aligns with earlier reports that antigen-specific TCR transcripts are very low in frequency or under the detection limit in tumor tissue [40], suggesting that our expansion protocol favors outgrowth of T-cell clones which have not yet been expanded in vivo. While traditional TIL expansion protocols using a high-dose of IL-2 often result in significant contraction of the TCR repertoire due to the rapid dominance of effector clones [41], our sequential use of IL-7, IL-15, and IL-21 followed by IL-2, IL-7, and IL-15 in the presence of CL maintains a highly diverse population. Indeed, this diverse repertoire ensures that the final cell product retains the capacity to recognize a broader array of TAAs, such as the specific KRAS epitopes identified in our functional assays.

We describe here recognition of an HLA-DP–restricted target antigen originating from mitochondria. Non-lipid mitochondrial proteins have been reported to be naturally processed and loaded onto MHC class I molecules, *e.g.,* onto HLA-B*1801 [42]. Although mitochondria-derived peptides have been eluted from MHC molecules, it has, to our knowledge, not been described that such peptides are recognized by T-cells. We show here that the peptide FVSSMSASAVVLALAKIEIKLSD generated from a mutation in the ubiquitin–cytochrome C reductase Rieske iron-sulphur polypeptide 1 (UQCRFS1) protein is recognized by TIL and restricted by HLA-DP*04:01. Notably, autologous tumor recognition was observed not only in the bulk TIL line, but also in the UQCRFS1 peptide–expanded oligoclonal TIL line. This recognition was specifically inhibited by an anti-DP antibody, whereas no blocking was observed with control anti-DR or MHC class I antibodies (Figure 5E). UQCRFS1 belongs to the mitochondrial electron respiratory chain complex III (there are four enzyme complexes, designated as CI-CIV), with elevated protein expression in gastric cancer and breast adenocarcinoma [43,44]. While mitochondrial mutations were historically dismissed as infrequent, recent pan-cancer analyses have demonstrated that the majority of solid tumors harbor non-synonymous mtDNA mutations, particularly within the coding regions of OXPHOS (oxidative phosphorylation) complex subunits [45]. Due to the high copy number of mitochondrial genomes (heteroplasmy), these mutations can lead to increased neoantigen presentation compared to single-copy nuclear mutations [46]. This therapeutic potential is supported by Pierini et al. (2015), who demonstrated in a murine renal adenocarcinoma model that vaccination—using either dendritic cells pulsed with mitochondrial lysates or specific peptides derived from mitochondrial-encoded genes—elicited a potent cytotoxic T-cell response [47]. Biologically relevant immune responses were also observed using purified mitochondria from a melanoma cell line as a vaccine in a murine model [48], while CD4+ T-cells from patients with melanoma have been described to recognize mutant mitochondrial target epitopes [49]. The identification of a mutation in UQCRFS1—a core component of the OXPHOS machinery—in this report lends further support to the hypothesis that dysregulated mitochondrial protein expression or mitochondrial point mutations are subject to immune surveillance [45]. UQCRFS1 overexpression leads to apoptosis, DNA damage, and oxidative phosphorylation [44], whereas downregulation is associated with *alopecia totalis* and cardiomyopathy [50]. More research is needed to examine the role of glycolipids or lipid–receptor complexes as targets for anti-cancer directed immune responses. Of note, the low-density lipoprotein receptor–related protein 1B (LRP1B), which has also been shown to be recognized by TIL, is a member of the LDL receptor family and to be one of the most frequently altered cancer-associated genes [51]. The mutant LRP1B peptide–expanded oligoclonal TIL line reacted to the mutant, yet not the wildtype peptide, and also recognized autologous tumor cells (Figure 5E), providing further evidence that this peptide is naturally processed and presented.

One may speculate whether CL serves as a pathogen-associated molecular pattern (PAMP) stimulator [52,53], which may be derived not only from the inner mitochondrial membrane, but also by closely related phospholipid species in bacteria and fungi [14,54], some of which are associated with increased survival in patients with pancreatic cancer [55,56]. To summarize, we identified a distinct TIL population which may survey mitochondrial protein antigens. Upon completion of the current study, a different report [57] showed the transfer of mitochondria from immune cells to cancer cells and vice versa, with mitochondria extracted from TIL exhibiting the same mutations as those in cancer cells. In general, the exchange of mitochondria between cells has been reported before [58], which may imply the transfer of mutant mitochondrial proteins recognized by TIL that are presented either by cancer cells or by recipient cells in the tumor microenvironment receiving mutant mitochondria.

## 5. Conclusions

We reported the effective expansion of TIL from patients with epithelial cancer by adding cardiolipin. CL-expanded TIL expressed CXCL9/CXCL10 and recognized a broad panel of shared MHC class I– or class II–presented specific cancer target epitopes. TIL exhibited a broad TCR repertoire and contained oligoclonal T-cells recognizing an HLA-DP–restricted mutant target epitope derived from a mitochondria-associated protein presented by autologous tumor cells, suggesting that mitochondrial mutations undergo immune surveillance.

## Data Availability

RNA-Seq and AIRR-Seq data that support the findings of this study have been deposited in EBI European Nucleotide Archive with the primary accession code PRJEB64410. The exome and spatial transcriptomics data that support the findings of this study are available on reasonable request from the corresponding author; these data are not publicly available due to the sensitive nature of the data sets.

## Supplementary Materials

The following supporting information can be downloaded at: https://www.mdpi.com/article/10.3390/cells15090798/s1, Figure S1: Dose titration, cellular viability, and cytokine secretome profiling induced by Cardiolipin; Figure S2: Immunophenotypic analysis of TIL expanded in the presence of absence of Cardiolipin (275 nM); Figure S3: No difference in functional TIL profiles expanded with and without CL (275 nM); Figure S4: Gene expression map in T-cell activation/exhaustion markers and tissue homing [59,60,61]; Table S1: Overview of tumor histologies, TIL, tumor microfragments and PBMC analysis; Table S2: Overview of patient diagnosis and treatment history prior to PBMC collection; Table S3: KRAS peptides used as targets in the MCC950 assay and in the siRNA NLRP3 assay. In red marked point mutations as compared to the wt protein; Table S4: Number of distinct TCR CDR3 Sequences; Table S5: Sum of the percentages of the Top 10 CDR3 clonotype sequences for the TCR VA and VB chains in TIL or in tumor tissue (D1309, D1313, D1317, D1601 and D1607); Table S6: Commonly shared tumor-associated targets to gauge TIL reactivity. In red point mutations as compared to the wt protein; Table S7: IFN-γ production (pg/mL) in TIL against shared tumor-associated target antigens (TIL patients D1313, D1309, D3731 and D1688); Table S8: D1309 neoepitopes (15 mer) derived from DNA exome sequencing. Red: marked point mutation as compared to the wt protein; Table S9: D1313 neoepitopes (15 mer, unless fitted to HLA molecules) derived from DNA exome sequencing derived. Red: marked point mutation as compared to the wt protein; Table S10: D3731 neoepitopes (15 mer, unless fitted to HLA molecules) derived from DNA exome sequencing. Red: marked point mutation as compared to the wt protein; Table S11: D1688 neoepitopes (15 mer, unless fitted to HLA molecules) derived from DNA exome sequencing. Red: marked point mutation as compared to the wt protein; Table S12: Top 10 CDR3 sequences for the TCR VA and VB chain in TIL expanded with the DPB1*04:02_UQCRFS1 peptide (FVSSMSASAVVLALAKIEIKLSD) from patient D1313; Table S13: CDR3 sequences for the TCR VA and VB chains in TIL expanded with Mesothelin_Pos106-120_WT (HRLSEPPEDLDALPL) peptide from D1313; Table S14: Top 10 CDR3 sequences for the TCR VA and VB chain in TIL expanded with the LRP1B peptide (VSKRLKFSRDLSLDP) from Patient D1309.

## References

[1] Rosenberg S.A., Yang J.C., Sherry R.M., Kammula U.S., Hughes M.S., Phan G.Q., Citrin D.E., Restifo N.P., Robbins P.F., Wunderlich J.R. (2011). Durable complete responses in heavily pretreated patients with metastatic melanoma using T-cell transfer immunotherapy. Clin. Cancer Res..

[2] Stevanovic S., Helman S.R., Wunderlich J.R., Langhan M.M., Doran S.L., Kwong M.L.M., Somerville R.P.T., Klebanoff C.A., Kammula U.S., Sherry R.M. (2019). A Phase II Study of Tumor-infiltrating Lymphocyte Therapy for Human Papillomavirus-associated Epithelial Cancers. Clin. Cancer Res..

[3] Creelan B.C., Wang C., Teer J.K., Toloza E.M., Yao J., Kim S., Landin A.M., Mullinax J.E., Saller J.J., Saltos A.N. (2021). Tumor-infiltrating lymphocyte treatment for anti-PD-1-resistant metastatic lung cancer: A phase 1 trial. Nat. Med..

[4] Zacharakis N., Chinnasamy H., Black M., Xu H., Lu Y.C., Zheng Z., Pasetto A., Langhan M., Shelton T., Prickett T. (2018). Immune recognition of somatic mutations leading to complete durable regression in metastatic breast cancer. Nat. Med..

[5] Tran E., Turcotte S., Gros A., Robbins P.F., Lu Y.C., Dudley M.E., Wunderlich J.R., Somerville R.P., Hogan K., Hinrichs C.S. (2014). Cancer immunotherapy based on mutation-specific CD4+ T cells in a patient with epithelial cancer. Science.

[6] Tran E., Robbins P.F., Lu Y.C., Prickett T.D., Gartner J.J., Jia L., Pasetto A., Zheng Z., Ray S., Groh E.M. (2016). T-Cell Transfer Therapy Targeting Mutant KRAS in Cancer. N. Engl. J. Med..

[7] Rauch J., Tannenbaum H., Stollar B.D., Schwartz R.S. (1984). Monoclonal anti-cardiolipin antibodies bind to DNA. Eur. J. Immunol..

[8] Reinstein E., Shoenfeld Y. (2007). Antiphospholipid syndrome and cancer. Clin. Rev. Allergy Immunol..

[9] Gomez-Puerta J.A., Cervera R., Espinosa G., Aguilo S., Bucciarelli S., Ramos-Casals M., Ingelmo M., Asherson R.A., Font J. (2006). Antiphospholipid antibodies associated with malignancies: Clinical and pathological characteristics of 120 patients. Semin. Arthritis Rheum..

[10] Abdel-Wahab N., Tayar J.H., Fa’ak F., Sharma G., Lopez-Olivo M.A., Yousif A., Shagroni T., Al-Hawamdeh S., Rojas-Hernandez C.M., Suarez-Almazor M.E. (2020). Systematic review of observational studies reporting antiphospholipid antibodies in patients with solid tumors. Blood Adv..

[11] Jacome-Sosa M., Miao Z.F., Peche V.S., Morris E.F., Narendran R., Pietka K.M., Samovski D., Lo H.G., Pietka T., Varro A. (2021). CD36 maintains the gastric mucosa and associates with gastric disease. Commun. Biol..

[12] Cho J.A., Kim T.J., Moon H.J., Kim Y.J., Yoon H.K., Seong S.Y. (2018). Cardiolipin activates antigen-presenting cells via TLR2-PI3K-PKN1-AKT/p38-NF-kB signaling to prime antigen-specific naive T cells in mice. Eur. J. Immunol..

[13] Zhou R., Yazdi A.S., Menu P., Tschopp J. (2011). A role for mitochondria in NLRP3 inflammasome activation. Nature.

[14] Lerias J.R., de Sousa E., Paraschoudi G., Martins J., Condeco C., Figueiredo N., Carvalho C., Dodoo E., Maia A., Castillo-Martin M. (2019). Trained Immunity for Personalized Cancer Immunotherapy: Current Knowledge and Future Opportunities. Front. Microbiol..

[15] de Sousa E., Lerias J.R., Beltran A., Paraschoudi G., Condeco C., Kamiki J., Antonio P.A., Figueiredo N., Carvalho C., Castillo-Martin M. (2021). Targeting Neoepitopes to Treat Solid Malignancies: Immunosurgery. Front. Immunol..

[16] Fahrner J.E., Lahmar I., Goubet A.G., Haddad Y., Carrier A., Mazzenga M., Drubay D., Alves Costa Silva C., Lyon C.S.G., de Sousa E. (2022). The Polarity and Specificity of Antiviral T Lymphocyte Responses Determine Susceptibility to SARS-CoV-2 Infection in Patients with Cancer and Healthy Individuals. Cancer Discov..

[17] Jiang H., Lei R., Ding S.-W., Zhu S. (2014). Skewer: A fast and accurate adapter trimmer for next-generation sequencing paired-end reads. BMC Bioinform..

[18] Howe K.L., Achuthan P., Allen J., Allen J., Alvarez-Jarreta J., Amode M.R., Armean I.M., Azov A.G., Bennett R., Bhai J. (2021). Ensembl 2021. Nucleic Acids Res..

[19] Dobin A., Davis C.A., Schlesinger F., Drenkow J., Zaleski C., Jha S., Batut P., Chaisson M., Gingeras T.R. (2013). STAR: Ultrafast universal RNA-seq aligner. Bioinformatics.

[20] Love M.I., Huber W., Anders S. (2014). Moderated estimation of fold change and dispersion for RNA-seq data with DESeq2. Genome Biol..

[21] Li H., Durbin R. (2010). Fast and accurate long-read alignment with Burrows-Wheeler transform. Bioinformatics.

[22] Institute B. (2019). Picard Toolkit.

[23] McKenna A., Hanna M., Banks E., Sivachenko A., Cibulskis K., Kernytsky A., Garimella K., Altshuler D., Gabriel S., Daly M. (2010). The Genome Analysis Toolkit: A MapReduce framework for analyzing next-generation DNA sequencing data. Genome Res..

[24] de Sousa E., Lerias J.R., Gorgulho C.M., Chaves-Ferreira M., Balan V., Pan W., Byrne-Steele M., Wang Z., Han J., Gama-Carvalho M. (2026). TCRcloud: A global visualization tool for T-cell and B-cell receptor transcripts. J. Transl. Med..

[25] Cook M.E., Jarjour N.N., Lin C.C., Edelson B.T. (2020). Transcription Factor Bhlhe40 in Immunity and Autoimmunity. Trends Immunol..

[26] Meng Q., Liu Z., Rangelova E., Poiret T., Ambati A., Rane L., Xie S., Verbeke C., Dodoo E., Del Chiaro M. (2016). Expansion of Tumor-reactive T Cells From Patients With Pancreatic Cancer. J. Immunother..

[27] Klein K., He K., Younes A.I., Barsoumian H.B., Chen D., Ozgen T., Mosaffa S., Patel R.R., Gu M., Novaes J. (2020). Role of Mitochondria in Cancer Immune Evasion and Potential Therapeutic Approaches. Front. Immunol..

[28] Qian L., Yu S., Yin C., Zhu B., Chen Z., Meng Z., Wang P. (2019). Plasma IFN-gamma-inducible chemokines CXCL9 and CXCL10 correlate with survival and chemotherapeutic efficacy in advanced pancreatic ductal adenocarcinoma. Pancreatology.

[29] Nie S., Song Y., Hu K., Zu W., Zhang F., Chen L., Ma Q., Zhou Z., Jiao S. (2024). CXCL10 and IL15 co-expressing chimeric antigen receptor T cells enhance anti-tumor effects in gastric cancer by increasing cytotoxic effector cell accumulation and survival. Oncoimmunology.

[30] Xia M., Chen J., Meng G., Shen H., Dong J. (2021). CXCL10 encoding synNotch T cells enhance anti-tumor immune responses without systemic side effect. Biochem. Biophys. Res. Commun..

[31] Reschke R., Gajewski T.F. (2022). CXCL9 and CXCL10 bring the heat to tumors. Sci. Immunol..

[32] Dudley M.E., Wunderlich J.R., Shelton T.E., Even J., Rosenberg S.A. (2003). Generation of tumor-infiltrating lymphocyte cultures for use in adoptive transfer therapy for melanoma patients. J. Immunother..

[33] Hall M., Liu H., Malafa M., Centeno B., Hodul P.J., Pimiento J., Pilon-Thomas S., Sarnaik A.A. (2016). Expansion of tumor-infiltrating lymphocytes (TIL) from human pancreatic tumors. J. Immunother. Cancer.

[34] van der Burgh R., Boes M. (2015). Mitochondria in autoinflammation: Cause, mediator or bystander?. Trends Endocrinol. Metab..

[35] Kelley N., Jeltema D., Duan Y., He Y. (2019). The NLRP3 Inflammasome: An Overview of Mechanisms of Activation and Regulation. Int. J. Mol. Sci..

[36] Martinon F., Petrilli V., Mayor A., Tardivel A., Tschopp J. (2006). Gout-associated uric acid crystals activate the NALP3 inflammasome. Nature.

[37] Sutterwala F.S., Ogura Y., Szczepanik M., Lara-Tejero M., Lichtenberger G.S., Grant E.P., Bertin J., Coyle A.J., Galan J.E., Askenase P.W. (2006). Critical role for NALP3/CIAS1/Cryopyrin in innate and adaptive immunity through its regulation of caspase-1. Immunity.

[38] Rosenberg S.A., Anderson W.F., Blaese M., Hwu P., Yannelli J.R., Yang J.C., Topalian S.L., Schwartzentruber D.J., Weber J.S., Ettinghausen S.E. (1993). The development of gene therapy for the treatment of cancer. Ann. Surg..

[39] Poschke I.C., Hassel J.C., Rodriguez-Ehrenfried A., Lindner K.A.M., Heras-Murillo I., Appel L.M., Lehmann J., Lovgren T., Wickstrom S.L., Lauenstein C. (2020). The Outcome of Ex Vivo TIL Expansion Is Highly Influenced by Spatial Heterogeneity of the Tumor T-Cell Repertoire and Differences in Intrinsic In Vitro Growth Capacity between T-Cell Clones. Clin. Cancer Res..

[40] Lowery F.J., Krishna S., Yossef R., Parikh N.B., Chatani P.D., Zacharakis N., Parkhurst M.R., Levin N., Sindiri S., Sachs A. (2022). Molecular signatures of antitumor neoantigen-reactive T cells from metastatic human cancers. Science.

[41] Kongkaew T., Thaiwong R., Tudsamran S., Sae-Jung T., Sengprasert P., Vasuratna A., Suppipat K., Reantragoon R. (2022). TIL expansion with high dose IL-2 or low dose IL-2 with anti-CD3/anti-CD28 stimulation provides different quality of TIL-expanded T cell clones. J. Immunol. Methods.

[42] Hickman H.D., Luis A.D., Buchli R., Few S.R., Sathiamurthy M., VanGundy R.S., Giberson C.F., Hildebrand W.H. (2004). Toward a definition of self: Proteomic evaluation of the class I peptide repertoire. J. Immunol..

[43] Jun K.H., Kim S.Y., Yoon J.H., Song J.H., Park W.S. (2012). Amplification of the UQCRFS1 Gene in Gastric Cancers. J. Gastric Cancer.

[44] Sun Q., Li J., Dong H., Zhan J., Xiong X., Ding J., Li Y., He L., Wang J. (2023). UQCRFS1 serves as a prognostic biomarker and promotes the progression of ovarian cancer. Sci. Rep..

[45] Yuan Y., Ju Y.S., Kim Y., Li J., Wang Y., Yoon C.J., Yang Y., Martincorena I., Creighton C.J., Weinstein J.N. (2020). Comprehensive molecular characterization of mitochondrial genomes in human cancers. Nat. Genet..

[46] Grandhi S., Bosworth C., Maddox W., Sensiba C., Akhavanfard S., Ni Y., LaFramboise T. (2017). Heteroplasmic shifts in tumor mitochondrial genomes reveal tissue-specific signals of relaxed and positive selection. Hum. Mol. Genet..

[47] Pierini S., Fang C., Rafail S., Facciponte J.G., Huang J., De Sanctis F., Morgan M.A., Uribe-Herranz M., Tanyi J.L., Facciabene A. (2015). A Tumor Mitochondria Vaccine Protects against Experimental Renal Cell Carcinoma. J. Immunol..

[48] Prota G., Gileadi U., Rei M., Lechuga-Vieco A.V., Chen J.L., Galiani S., Bedard M., Lau V.W.C., Fanchi L.F., Artibani M. (2020). Enhanced Immunogenicity of Mitochondrial-Localized Proteins in Cancer Cells. Cancer Immunol. Res..

[49] Voo K.S., Zeng G., Mu J.B., Zhou J., Su X.Z., Wang R.F. (2006). CD4+ T-cell response to mitochondrial cytochrome B in human melanoma. Cancer Res..

[50] Gusic M., Schottmann G., Feichtinger R.G., Du C., Scholz C., Wagner M., Mayr J.A., Lee C.Y., Yepez V.A., Lorenz N. (2020). Bi-Allelic UQCRFS1 Variants Are Associated with Mitochondrial Complex III Deficiency, Cardiomyopathy, and Alopecia Totalis. Am. J. Hum. Genet..

[51] Principe C., Dionisio de Sousa I.J., Prazeres H., Soares P., Lima R.T. (2021). LRP1B: A Giant Lost in Cancer Translation. Pharmaceuticals.

[52] Janeway C.A. (1989). Approaching the asymptote? Evolution and revolution in immunology. Cold Spring Harb. Symp. Quant. Biol..

[53] Seong S.Y., Matzinger P. (2004). Hydrophobicity: An ancient damage-associated molecular pattern that initiates innate immune responses. Nat. Rev. Immunol..

[54] Rosa Ide A., Einicker-Lamas M., Bernardo R.R., Benchimol M. (2008). Cardiolipin, a lipid found in mitochondria, hydrogenosomes and bacteria was not detected in Giardia lamblia. Exp. Parasitol..

[55] Aykut B., Pushalkar S., Chen R., Li Q., Abengozar R., Kim J.I., Shadaloey S.A., Wu D., Preiss P., Verma N. (2019). The fungal mycobiome promotes pancreatic oncogenesis via activation of MBL. Nature.

[56] Riquelme E., Zhang Y., Zhang L., Montiel M., Zoltan M., Dong W., Quesada P., Sahin I., Chandra V., San Lucas A. (2019). Tumor Microbiome Diversity and Composition Influence Pancreatic Cancer Outcomes. Cell.

[57] Ikeda H., Kawase K., Nishi T., Watanabe T., Takenaga K., Inozume T., Ishino T., Aki S., Lin J., Kawashima S. (2025). Immune evasion through mitochondrial transfer in the tumour microenvironment. Nature.

[58] Saha T., Dash C., Jayabalan R., Khiste S., Kulkarni A., Kurmi K., Mondal J., Majumder P.K., Bardia A., Jang H.L. (2022). Intercellular nanotubes mediate mitochondrial trafficking between cancer and immune cells. Nat. Nanotechnol..

[59] Tirosh I., Izar B., Prakadan S.M., Wadsworth M.H., Treacy D., Trombetta J.J., Rotem A., Rodman C., Lian C., Murphy G. (2016). Dissecting the multicellular ecosystem of metastatic melanoma by single-cell RNA-seq. Science.

[60] Siddiqui I., Schaeuble K., Chennupati V., Fuertes Marraco S.A., Calderon-Copete S., Pais Ferreira D., Carmona S.J., Scarpellino L., Gfeller D., Pradervand S. (2019). Intratumoral Tcf1(+)PD-1(+)CD8(+) T Cells with Stem-like Properties Promote Tumor Control in Response to Vaccination and Checkpoint Blockade Immunotherapy. Immunity.

[61] Chen Y., Shen J., Kasmani M.Y., Topchyan P., Cui W. (2021). Single-Cell Transcriptomics Reveals Core Regulatory Programs That Determine the Heterogeneity of Circulating and Tissue-Resident Memory CD8(+) T Cells. Cells.

