# Supplementary material for: Cardiolipin Induces CXCL9/CXCL10 Expression in Tumor-Infiltrating Lymphocytes
Source: Cells. 2026 Apr 28;15(9):798. doi: 10.3390/cells15090798 (PMC13162724; doi:10.3390/cells15090798)
Supplement: Supplementary file 1 [file cells-15-00798-s001.zip › cells-4106197-supplementary.pdf]

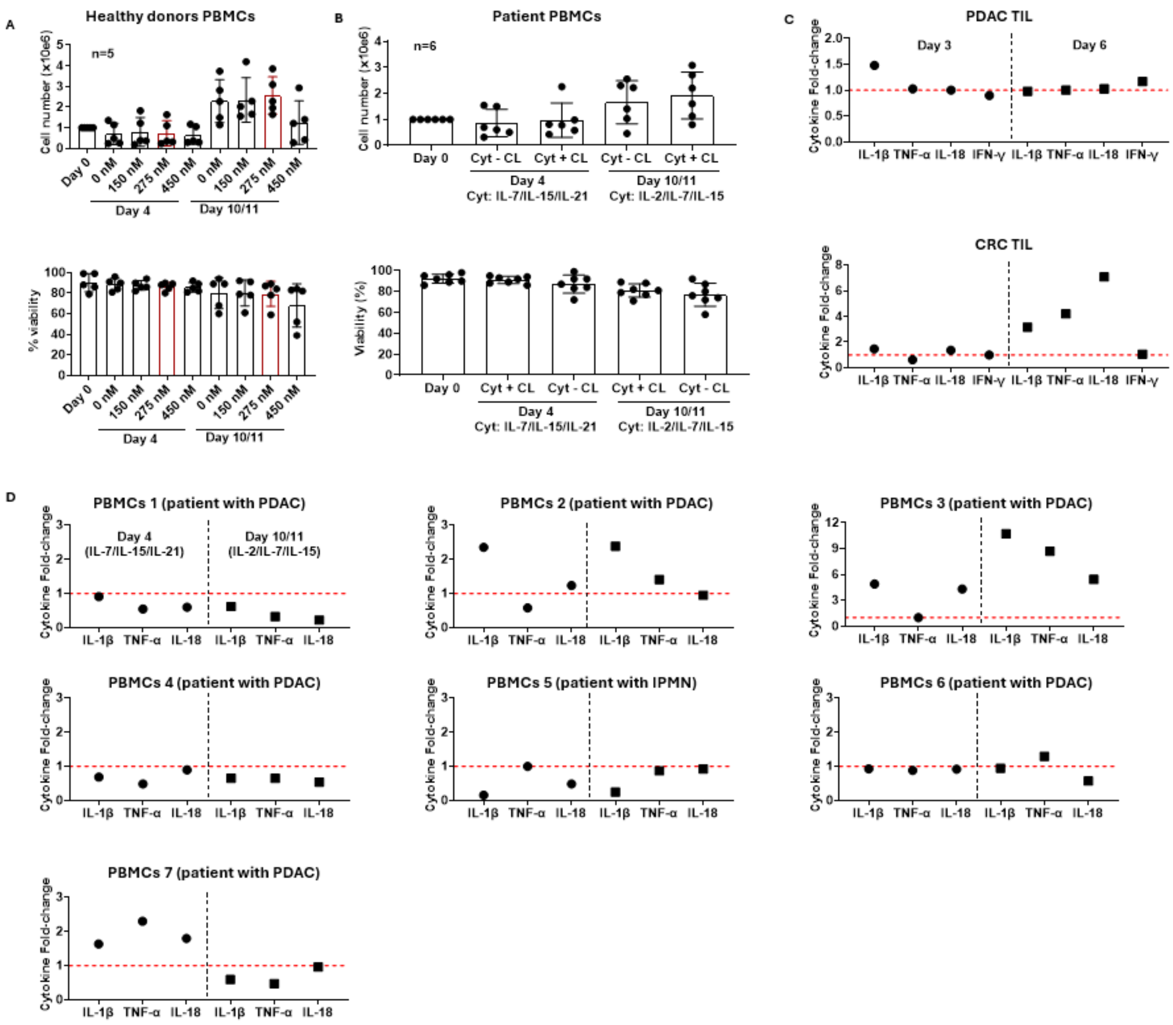


Supplementary Figure S1. Dose titration, cellular viability, and cytokine secretome profiling induced by Cardiolipin (A) PBMCs from healthy donors were cultured with different concentrations of CL (0 to 450 nM). The top panel shows absolute cell counts, and the bottom panel displays cellular viability (determined by Trypan Blue exclusion), demon-strating a lack of toxicity at the selected working dose of 275 nM; (B) Assessment of cell expansion (top panel) and via-bility (bottom panel) in PBMCs isolated from patients, cultured with cytokine cocktail in the presence or absence of 275 nM CL; (C) Secretion levels of TNF-$\alpha$, IL-1β, IL-18, and IFN-$\gamma$ measured in TIL cell culture supernatants (n=2) with or without CL supplementation. CL treatment enhanced the production of both effector cytokines and inflam-masome-related mediators; (D) Secretome analysis of patient-derived PBMCs cultured with the cytokine cocktail used for the initial TIL expansion (IL-7, IL-15, IL-21) with or without CL. The addition of CL induced an increase TNF-$\alpha$, IL-1β and IL-18, production, consistent with cytokines associated with the NLRP3-pathway.

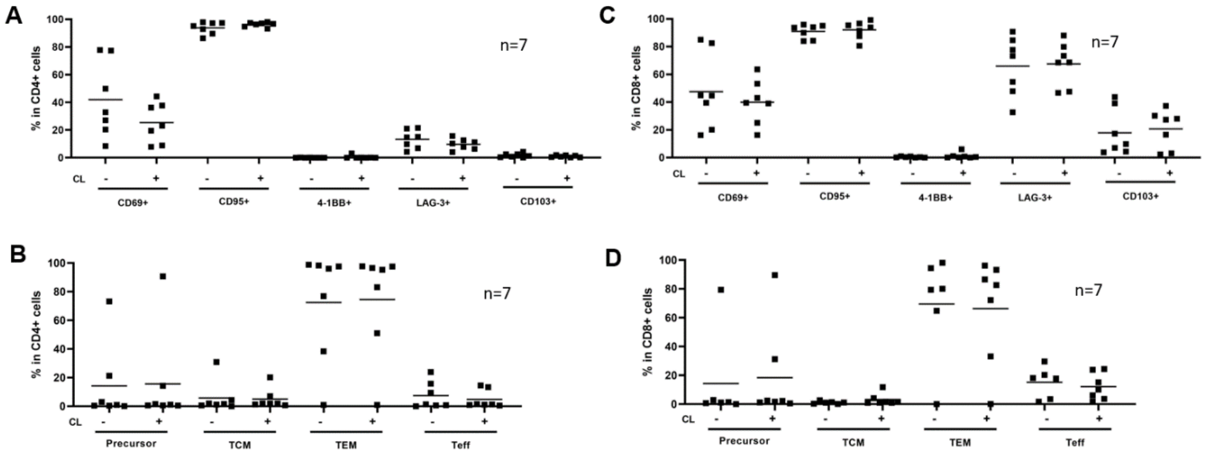


Supplementary Figure S2. Immunophenotypic analysis of TIL expanded in the presence of absence of Cardiolipin (275 nM) (A, B, C, D) Markers associated with exhaustion/activation of T-cells were analyzed in TIL expanded in the presence or absence of CL, i.e., in CD4+ T-cells (A) and CD8+ T-cells (B), showing no differences in regard CD4/CD8 frequencies. T-cell memory phenotype was also analyzed using CD45RA/CCR7 markers, examining precursor cells, central memory T-cells (TCM), effector memory T-cells (TEM) and T-effector cells (Teff) in CD4+ T-cells (C) and CD8+ T-cells (D); no differences in CD45RA/CCR7 frequencies. Each dot corresponds to TIL from a different patient.

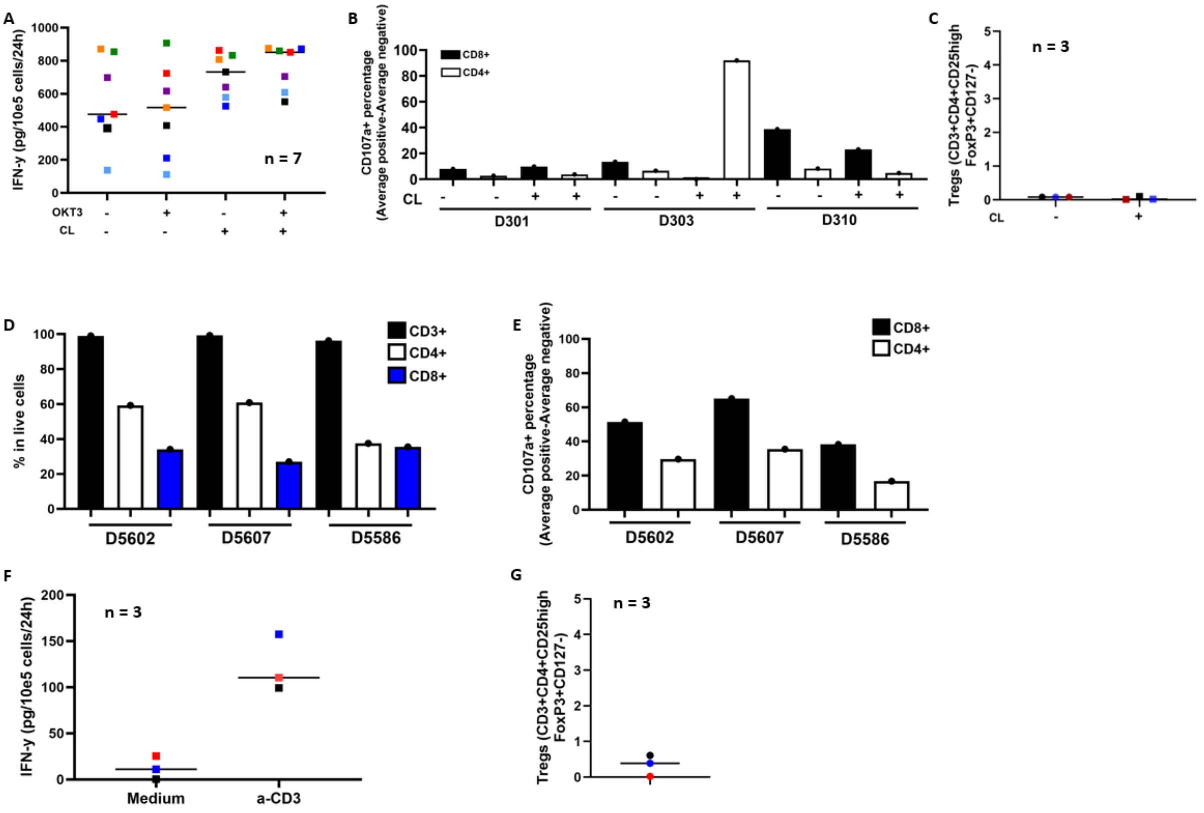


Supplementary Figure S3. No difference in functional TIL profiles expanded with and without CL (275nM) (A) TIL expanded with or without CL were tested using a standard 24-hour CD3 cross-linking assay using OKT3 (30 ng/mL); for cytotoxicity measured by an CD107a assay (B) or for Tregs flow cytometric analysis (C); TIL expanded with non-IgG coupled cytokines were tested for immunophenotype (D), for cytotoxicity measured by an CD107a assay (E), or were tested using a standard 24-hour CD3 cross-linking assay using anti-CD3 (30 ng/mL) (F) or for Tregs flow cytometric analysis (G).

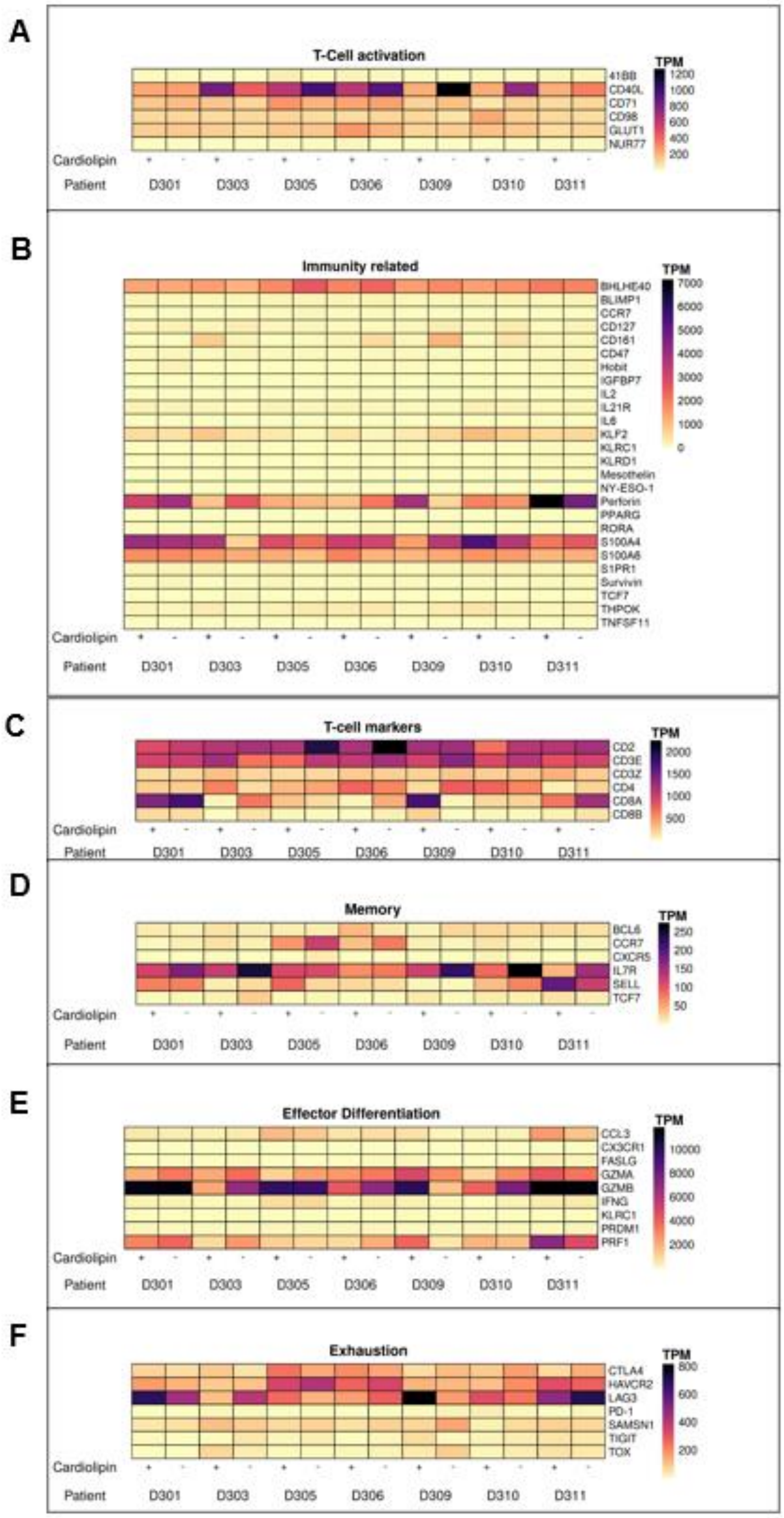


Supplementary Figure S4. Gene expression map in T-cell activation/exhaustion markers and tissue homing (A, B, C, D, E, F) RNA sequencing was performed in TIL expanded with or without CL and reads were normalized. Gene expression associated with (A) T-cell activation, (B) immunity, (C) T-cell marker, (D) memory phenotype, (E) effector differentiation phenotype and (F) exhaustion phenotype is shown with no clear differences between CL-/+ groups. RNA expression profiles were chosen based on publications [59-61].

Supplementary Table S1. Overview of tumor histologies, TIL, tumor microfragments and PBMC analysis

| Sample ID | Histology | Assays Performed |
|---|---|---|
| D1309 | PDAC | T-cells tumor recognition<br>DNA exome sequencing<br>TCR sequencing<br>Neoepitope assays |
| D1317 | PDAC | T-cell tumor recognition |
| D3270 | Duodenum adenocarcinoma | αβ T-cell tumor recognition |
| D1313 | NET | αβ T-cells tumor recognition<br>unsorted T-cell tumor recognition<br>DNA Exome sequencing<br>TCR sequencing<br>Neoepitope assays<br>Spatial transcriptomics |
| D1209 | PDAC | αβ T-cell tumor recognition |
| D1225 | Colon adenocarcinoma metastatic to liver | αβ T-cell tumor recognition |
| D3276 | Colon adenocarcinoma metastatic to liver | αβ T-cell tumor recognition |
| D1130 | Cystic Teratoma | αβ T-cell Tumor recognition |
| D1688 | Intrahepatic cholangiocarcinoma | DNA exome sequencing<br>TCR sequencing<br>Neoepitope assays<br>Spatial transcriptomics<br>TAAT recognition assay |
| D3731 | PDAC | DNA exome sequencing<br>TCR sequencing<br>Neoepitope assays<br>TAAT recognition assay |
| D1601 | PDAC | TCR sequencing<br>TAAT recognition assay |
| D1607 | NET | TCR sequencing<br>TAAT recognition assay |
| D1211 | PDAC | MCC950– KRAS recognition |
| D1209 | PDAC | MCC950 assay – KRAS recognition |
| D1210 | PDAC metastatic to liver | MCC950 assay – KRAS recognition |
| D1316 | Retroperitoneal Sarcoma | MCC950 assay – KRAS recognition |
| D1317 | PDAC | MCC950 assay – KRAS recognition |
| D1319 | Liposarcoma | MCC950 assay – KRAS recognition |
| D1320 | Gastric cancer | MCC950 assay – KRAS recognition |
| D301 | NET | RNA Sequencing of expanded TIL |
| D303 | Colorectal adenocarcinoma | RNA Sequencing of expanded TIL |
| D305 | NET | RNA Sequencing of expanded TIL |
| D306 | PDAC | RNA Sequencing of expanded TIL |
| D309 | PDAC | RNA Sequencing of expanded TIL |
| D310 | Colorectal adenocarcinoma | RNA Sequencing of expanded TIL |

| | | |
|---|---|---|
| D311 | Colorectal adenocarcinoma | RNA Sequencing of expanded TIL |
| D5780 | PDAC | PBMCs secretome analysis |
| D5781 | PDAC | PBMCs secretome analysis |
| D5782 | PDAC | PBMCs secretome analysis<br>TIL secretome analysis<br>Tumor microfragments secretome and CXCL10 analysis |
| D5789 | PDAC | PBMCs secretome analysis |
| D5793 | IPMN | PBMCs secretome analysis |
| D5796 | PDAC | PBMCs secretome analysis |
| D5825 | PDAC | PBMCs secretome analysis |
| D5797 | CRC | Tumor microfragments secretome and CXCL10 analysis |
| D5801 | PDAC metastatic to liver | Tumor microfragments secretome and CXCL10 analysis |
| D5836 | Ascendant colon adenocarcinoma | Tumor microfragments secretome and CXCL10 analysis |
| D5841 | PDAC | siRNA NLRP3 assay |
| D5602 | PDAC | Non-IgG fused cytokine assay |
| D5607 | PDAC | Non-IgG fused cytokine assay |
| D5586 | PDAC | Non-IgG fused cytokine assay |

Immunohistochemistry (IHC), Pancreatic ductal adenocarcinoma (PDAC), Neuroendocrine pancreatic tumor (NET), colon adenocarcinoma (CRC), Intraductal Papillary Mucinous Neoplasia (IPMN).

Supplementary Table S2. Overview of patient diagnosis and treatment history prior to PBMC collection.

| Sample ID | Histology | Treatment Regimen | Number of cycles |
| --- | --- | --- | --- |
| PBMCs 1 | PDAC | FOLFIRINOX | 18 |
| PBMCs 2 | PDAC | FOLFIRINOX | 6 |
| PBMCs 3 | PDAC | FOLFIRINOX | 10 |
| PBMCs 4 | PDAC | No chemotherapy | N/A |
| PBMCs 5 | IPMN | No chemotherapy | N/A |
| PBMCs 6 | PDAC | No chemotherapy | N/A |
| PBMCs 7 | PDAC | mFOLFIRINOX | 8 |

Supplementary Table S3. KRAS peptides used as targets in the MCC950 assay and in the siRNA NLRP3 assay. In red marked point mutations as compared to the wt protein

| Peptide ID | Peptide sequence |
|---|---|
| KRAS_Pos4-20_WT | YKLVVVGAGGVGKSALT |
| KRAS_Pos4-20_G12V | YKLVVVGAVGVGKSALT |
| KRAS_Pos4-20_G12D | YKLVVVGADGVGKSALT |
| KRAS_Pos4-20_G12R | YKLVVVGARGVGKSALT |
| KRAS_Pos4-20_G12S | YKLVVVGASGVGKSALT |
| KRAS_Pos4-20_G12C | YKLVVVGACGVGKSALT |
| KRAS_Pos4-20_G12A | YKLVVVGAAGVGKSALT |
| KRAS_Pos5-21_WT | KLVVVGAGGVGKSALTI |
| KRAS_Pos5-21_G13D | KLVVVGAGDVGKSALTI |
| KRAS_Pos53-69_WT | LDILDTAGQEEYSAMRD |
| KRAS_Pos53-69_Q61K | LDILDTAGKEEYSAMRD |
| KRAS_Pos53-69_Q61H | LDILDTAGHEEYSAMRD |
| KRAS_Pos53-69_Q61R | LDILDTAGREEYSAMRD |
| KRAS_Pos138-154_WT | GIPFIETSAKTRQRVED |
| KRAS_Pos138-154_A146T | GIPFIETSTKTRQRVED |
| KRAS_Pos5-19_G12F | KLVVVGAVGVGKSAL |
| KRAS_Pos6-20_G13C | LVVVGAGCVGKSALT |
| KRAS_Pos6-20_G13S | LVVVGAGSVGKSALT |
| KRAS_Pos6-20_G13R | LVVVGAGRVGKSALT |
| KRAS_Pos6-20_G13A | LVVVGAGAVGKSALT |
| KRAS_Pos6-20_G13V | LVVVGAGVVGKSALT |
| KRAS_Pos54-68_Q61L | DILDTAGLEEYSAMR |
| KRAS_Pos110-124_K117N | PMVLVGNNCDLPSRT |
| KRAS_Pos139-153_A146V | IPFIETSVKTRQRVE |
| KRAS_Pos6-20_G12V (fitted for HLA-DQB1*06:02) | LVVVGAVGVGKSALT |

Supplementary Table S4. Number of distinct TCR CDR3 Sequences

| | **TCR transcripts in tumor tissue** | **TIL** | **TIL stimulated with HLA-DPB1*04:02_UQCRFS1 peptide** | **TIL stimulated with Mesothelin_Pos106-120_WT peptide** | **TIL stimulated with LRP1B peptide** |
|---|---|---|---|---|---|
| **D1309 α-chain** | 930 | 2392 | - | - | 308 |
| **D1309 β-chain** | 2538 | 2465 | - | - | 188 |
| **D1313 α-chain** | 1663 | 3828 | 15 | 4 | - |
| **D1313 β-chain** | 2889 | 3655 | 11 | 5 | - |
| **D1317 α-chain** | 31569 | 31666 | - | - | - |
| **D1317 β-chain** | 58872 | 40027 | - | - | - |
| **D1601 α-chain** | 14581 | 38797 | - | - | - |
| **D1601 β-chain** | 30223 | 39913 | - | - | - |
| **D1607 α-chain** | 1032 | 11223 | - | - | - |
| **D1607 β-chain** | 2456 | 11878 | - | - | - |

Supplementary Table S5. Sum of the percentages of the Top 10 CDR3 clonotype sequences for the TCR VA and VB chains in TIL or in tumor tissue (D1309, D1313, D1317, D1601 and D1607)

| **TCR transcripts** | **Alpha chain** | | **Beta chain** | |
|---|---|---|---|---|
| | **Tumor** | **TIL** | **Tumor** | **TIL** |
| **D1309** | 8.69% | 40.00% | 10.29% | 29.40% |
| **D1313** | 10.68% | 98.21% | 15.82% | 96.39% |
| **D1317** | 14.96% | 25.03% | 16.58% | 8.87% |
| **D1601** | 15.51% | 10.71% | 12.03% | 7.85% |
| **D1607** | 8.23% | 25.34% | 6.32% | 36.97% |
| **Average %** | 11.61% | 39.86% | 12.21% | 35.90% |

Supplementary Table S6. Commonly shared tumor-associated targets to gauge TIL reactivity. In red point mutations as compared to the wt protein

| Target | Peptide ID | Peptide sequence |
|---|---|---|
| KRAS | KRAS_Pos4-20_WT | YKLVVVGAGGVGKSALT |
| KRAS | KRAS_Pos4-20_G12V | YKLVVVGAVGVGKSALT |
| KRAS | KRAS_Pos4-20_G12D | YKLVVVGADGVGKSALT |
| KRAS | KRAS_Pos4-20_G12R | YKLVVVGARGVGKSALT |
| KRAS | KRAS_Pos4-20_G12S | YKLVVVGASGVGKSALT |
| KRAS | KRAS_Pos4-20_G12C | YKLVVVGACGVGKSALT |
| KRAS | KRAS_Pos4-20_G12A | YKLVVVGAAGVGKSALT |
| KRAS | KRAS_Pos5-21_WT | KLVVVGAGGVGKSALTI |
| KRAS | KRAS_Pos5-21_G13D | KLVVVGAGDVGKSALTI |
| KRAS | KRAS_Pos53-69_WT | LDILDTAGQEEYSAMRD |
| KRAS | KRAS_Pos53-69_Q61K | LDILDTAGKEEYSAMRD |
| KRAS | KRAS_Pos53-69_Q61H | LDILDTAGHEEYSAMRD |
| KRAS | KRAS_Pos53-69_Q61R | LDILDTAGREEYSAMRD |
| KRAS | KRAS_Pos138-154_WT | GIPFIETSAKTRQRVED |
| KRAS | KRAS_Pos138-154_A146T | GIPFIETSTKTRQRVED |
| KRAS | KRAS_Pos5-19_G12F | KLVVVGAVGVGKSAL |
| KRAS | KRAS_Pos6-20_G13C | LVVVGAGCVGKSALT |
| KRAS | KRAS_Pos6-20_G13S | LVVVGAGSVGKSALT |
| KRAS | KRAS_Pos6-20_G13R | LVVVGAGRVGKSALT |
| KRAS | KRAS_Pos6-20_G13A | LVVVGAGAVGKSALT |
| KRAS | KRAS_Pos6-20_G13V | LVVVGAGVVGKSALT |
| KRAS | KRAS_Pos54-68_Q61L | DILDTAGLEEYSAMR |
| KRAS | KRAS_Pos110-124_K117N | PMVLVGNNCDLPSRT |
| KRAS | KRAS_Pos139-153_A146V | IPFIETSVKTRQRVE |
| KRAS | KRAS_Pos6-20_G12V (fitted for HLA-DQB1*06:02) | LVVVGAVGVGKSALT |
| MUC4 | MUC4_Pos1055-1069_T1062A+WT | TPLPVTDASSASTGH |
| MUC4 | MUC4_Pos1055-1069_WT | TPLPVTDTSSASTGH |
| MUC4 | MUC4_Pos1074-1088_A1081V+WT | PVTSLSSVSTGDTTP |
| MUC4 | MUC4_Pos1074-1088_WT | PVTSLSSASTGDTTP |
| MUC4 | MUC4_Pos1934-1948_S1941I+WT | TTPLPVTIPSSASSG |
| MUC4 | MUC4_Pos1934-1948_WT | TTPLPVTSPSSASSG |
| MUC4 | MUC4_Pos1666-1680_A1673V | PVTSTSSVSTGHATP |
| MUC4 | MUC4_Pos1666-1680_WT | PVTSTSSASTGHATP |
| MUC4 | MUC4_Pos2210-2224_A2217E | PVTSPSSESTGHAIP |
| MUC4 | MUC4_Pos2210-2224_WT | PVTSPSSASTGHAIP |
| MUC4 | MUC4_Pos1615-1629_T1622N | TPLPVTDNSSASTGD |
| MUC4 | MUC4_Pos1615-1629_WT | TPLPVTDTSSASTGD |

| | | |
|---|---|---|
| MUC4 | MUC4_Pos1059-1069_T1062A+WT (fitted to HLA-A*01:01) | VTDTSSASTGH |
| MUC4 | MUC4_Pos1059-1069_WT (fitted to HLA-A*01:01) | VTDASSASTGH |
| Mesothelin | Mesothelin_Pos1-15_WT | MALPTARPLLGSCGT |
| Mesothelin | Mesothelin_Pos106-120_WT | HRLSEPPEDLDALPL |
| Mesothelin | Mesothelin_Pos151-165_WT | LLPRGAPERQRLLPA |
| Mesothelin | Mesothelin_Pos165-180_WT | ALACWGVRGSLLSEA |
| Mesothelin | Mesothelin_Pos196-210_WT | FVAESAEVLLPRLVS |
| Mesothelin | Mesothelin_Pos241-255_WT | SVSTMDALRGLLPVL |
| Mesothelin | Mesothelin_Pos586-600_WT | LQGGIPNGYLVLDLS |
| Mesothelin | Mesothelin_Pos601-615_WT | MQEALSGTPCLLGPG |
| Mesothelin | Mesothelin_Pos346_360_WT | YEQLDVLKHKLDELY |
| Mesothelin | Mesothelin_Pos458-472_WT | WAVRPQDLDTCDPRQ |
| Mesothelin | Mesothelin_Pos473-487_WT | LDVLYPKARLAFQNM |
| Mesothelin | Mesothelin_Pos286-630_Mix15mers | Peptide mix of Mesothelin peptides |
| Melan-A/MART-1 | MART-1_Pos26-35_A27L (fitted for HLA-A*02:01) | ELAGIGILTV |
| Melan-A/MART-1 | MART-1_Pos27-35_WT | AAGIGILTV |
| survivin | Survivin_Pos97-111_WT | TLGEFLKLDRERAKN |
| SMAD4 | SMAD4_Pos308-322_WT | LAFQPPISNHPAPEY |
| CMV | CMV | Peptide Pool of 138 15mers with 11 aa overlap of pp65 (UniProt ID: P06725) |
| EBNA3 | EBNA3 | Peptide Pool of 234 15mers with 11 aa overlap of Epstein-Barr nuclear antigen 3 (UniProt ID: P12977) |
| M1 | M1 | Peptide Pool of 61 15mers with 11 aa overlap of Matrix protein 1 (UniProt ID: B4UPA8) |

Supplementary Table S7. IFN-γ production (pg/mL) in TIL against shared tumor-associated target antigens (TIL patients D1313, D1309, D3731 and D1688)

| Protein | Peptide ID | D1309 (PDAC) | D1313 (NET) | D3731 (PDAC) | D1688 (ICC) |
|---|---|---|---|---|---|
| KRAS | KRAS_Pos4-20_WT | 124.9 | 0.0 | 0.0 | 0.0 |
| KRAS | KRAS_Pos4-20_G12V | 195.5 | 0.0 | 0.0 | 0.0 |
| KRAS | KRAS_Pos4-20_G12D | 0.0 | 0.0 | 0.0 | 0.0 |
| KRAS | KRAS_Pos4-20_G12R | 67.5 | 0.0 | 0.0 | 0.0 |
| KRAS | KRAS_Pos4-20_G12S | 86.4 | 0.0 | 0.0 | 0.0 |
| KRAS | KRAS_Pos4-20_G12C | 0.0 | 0.0 | 0.0 | 0.0 |
| KRAS | KRAS_Pos4-20_G12A | 54.6 | 0.0 | 0.0 | 0.0 |
| KRAS | KRAS_Pos5-21_WT | 191.1 | 0.0 | 0.0 | 0.0 |
| KRAS | KRAS_Pos5-21_G13D | 127.9 | 0.0 | 0.0 | 0.0 |
| KRAS | KRAS_Pos53-69_WT | 124.1 | 0.0 | 0.0 | 0.0 |
| KRAS | KRAS_Pos53-69_Q61K | 0.0 | 0.0 | 0.0 | 0.0 |
| KRAS | KRAS_Pos53-69_Q61H | 43.5 | 0.0 | 0.0 | 0.0 |
| KRAS | KRAS_Pos53-69_Q61R | 59.9 | 0.0 | 40.1 | 0.0 |
| KRAS | KRAS_Pos138-154_WT | 10.5 | 0.0 | 3.6 | 0.0 |
| KRAS | KRAS_Pos138-154_A146T | 0.0 | 0.0 | 0.0 | 0.0 |
| KRAS | KRAS_Pos5-19_G12F | 163.5 | 18.0 | 0.0 | 0.0 |
| KRAS | KRAS_Pos6-20_G13C | 143.0 | 21.5 | 17.9 | 0.0 |
| KRAS | KRAS_Pos6-20_G13S | 109.9 | 0.0 | 22.4 | 0.0 |
| KRAS | KRAS_Pos6-20_G13R | 265.4 | 0.0 | 37.8 | 0.0 |
| KRAS | KRAS_Pos6-20_G13A | 299.3 | 0.0 | 0.0 | 0.0 |
| KRAS | KRAS_Pos6-20_G13V | 94.1 | 2.9 | 0.0 | 0.0 |
| KRAS | KRAS_Pos54-68_Q61L | 88.8 | 0.0 | 0.0 | 0.0 |
| KRAS | KRAS_Pos110-124_K117N | 251.6 | 16.6 | 0.0 | 0.0 |
| KRAS | KRAS_Pos139-153_A146V | 91.8 | 0.0 | 0.0 | 0.0 |
| KRAS | KRAS_Pos6-20_G12V (fitted for HLA-DQB1*06:02) | 0.0 | 0.0 | 364.1 | 0.0 |
| MUC4 | MUC4_Pos1055-1069_T1062A+WT | 59.6 | 0.0 | 0.0 | 0.0 |
| MUC4 | MUC4_Pos1055-1069_WT | 0.0 | 0.0 | 0.0 | 0.0 |
| MUC4 | MUC4_Pos1074-1088_A1081V+WT | 67.2 | 0.0 | 0.0 | 0.0 |
| MUC4 | MUC4_Pos1074-1088_WT | 91.4 | 0.0 | 28.6 | 0.0 |
| MUC4 | MUC4_Pos1934-1948_S1941I+WT | 0.0 | 0.0 | 0.0 | 0.0 |
| MUC4 | MUC4_Pos1934-1948_WT | 0.0 | 0.0 | 0.0 | 0.0 |
| MUC4 | MUC4_Pos1666-1680_A1673V | 0.0 | 0.0 | 0.0 | 0.0 |
| MUC4 | MUC4_Pos1666-1680_WT | 0.0 | 0.0 | 0.0 | 0.0 |
| MUC4 | MUC4_Pos2210-2224_A2217E | 1.3 | 0.0 | 0.0 | 0.0 |
| MUC4 | MUC4_Pos2210-2224_WT | 0.0 | 0.0 | 0.0 | 0.0 |
| MUC4 | MUC4_Pos1615-1629_T1622N | 12.3 | 0.0 | 0.0 | 0.0 |

| | | | | | |
|---|---|---|---|---|---|
| MUC4 | MUC4_Pos1615-1629_WT | 129.1 | 0.0 | 110.1 | 0.0 |
| MUC4 | MUC4_Pos1059-1069_T1062A+WT (fitted for HLA-A*01:01) | 246.2 | 0.0 | 0.0 | 0.0 |
| MUC4 | MUC4_Pos1059-1069_WT (fitted for HLA-A*01:01) | 74.9 | 0.0 | 0.0 | 0.0 |
| antigen | Mesothelin_Pos1-15_WT | 47.9 | 2.1 | 0.0 | 0.0 |
| antigen | Mesothelin_Pos106-120_WT | 70.3 | 745.9 | 0.0 | 0.0 |
| antigen | Mesothelin_Pos151-165_WT | 5.8 | 10.4 | 0.0 | 0.0 |
| antigen | Mesothelin_Pos165-180_WT | 121.3 | 48.9 | 10.1 | 0.0 |
| antigen | Mesothelin_Pos196-210_WT | 47.6 | 0.0 | 103.5 | 0.0 |
| antigen | Mesothelin_Pos241-255_WT | 0.0 | 0.0 | 0.0 | 0.0 |
| antigen | Mesothelin_Pos586-600_WT | 119.6 | 0.0 | 0.0 | 0.0 |
| antigen | Mesothelin_Pos601-615_WT | 176.6 | 0.0 | 0.0 | 0.0 |
| antigen | Mesothelin_Pos346_360_WT | 81.7 | 0.0 | 0.0 | 0.0 |
| antigen | Mesothelin_Pos458-472_WT | 34.9 | 0.0 | 0.0 | 0.0 |
| antigen | Mesothelin_Pos473-487_WT | 118.0 | 0.0 | 0.0 | 0.0 |
| antigen | Mesothelin_Pos286-630_Mix15mers | 84.5 | 14.2 | 26.0 | 0.0 |
| antigen | MART-1_Pos26-35_A27L (fitted for HLA-A*02:01) | 0.0 | 0.0 | 0.0 | 0.0 |
| antigen | MART-1_Pos27-35_WT | 142.7 | 0.0 | 0.0 | 0.0 |
| antigen | Survivin_Pos97-111_WT | 60.6 | 0.0 | 0.0 | 0.0 |
| antigen | SMAD4_Pos308-322_WT | 130.3 | 47.1 | 0.0 | 0.0 |
| positive control | PHA | 1496.0 | 1016.9 | 177.2 | 1602.2 |
| positive control | Anti-CD3 | 822.5 | 126.5 | 213.4 | 419.8 |
| positive control | Peptide Pool of 138 15mers with 11 aa overlap of pp65 (UniProt ID: P06725) | 211.2 | 0.0 | 0.0 | 0.0 |
| positive control | Peptide Pool of 234 15mers with 11 aa overlap of Epstein-Barr nuclear antigen 3 (UniProt ID: P12977) | 240.3 | 0.0 | 15.2 | 0.0 |
| positive control | Peptide Pool of 61 15mers with 11 aa overlap of Matrix protein 1 (UniProt ID: B4UPA8) | 0.0 | 0.0 | 57.2 | 0.0 |

Pancreatic ductal adenocarcinoma (PDAC), Neuroendocrine pancreatic tumor (NET); Intrahepatic cholangiosarcoma (ICC).

Supplementary Table S8. D1309 neoepitopes (15mer) derived from DNA exome sequencing. Red: marked point mutation as compared to the wt protein

| Name | Peptide | Mutation |
|---|---|---|
| LRP1B | VSKRLKFSRDLSLDP | c.9394C>T:p.Pro3132Ser |
| ALMS1 | YSHTEKPDVFYQQVL | c.4019G>A:p.Gly1340Asp |
| CDH12 | PVGTIIGVVTAQDLD | c.1214C>T:p.Ala405Val |
| GLIS3 | IGKGSCNSLVVTSSP | c.443A>G:p.Asn148Ser |
| HTR1E | LDRYWAISNAIEYAR | c.374C>G:p.Thr125Ser |
| MYT1L | PIAAAEKMAKAQEKH | c.1738C>A:p.Leu580Met |
| ONECUT2 | TLTPLQPQPPISTVS | c.470T>A:p.Leu157Gln |
| WFS1 | SMVKLILLWLTAIVL | c.1912G>T:p.Val638Leu |
| LRP1B_wt | VSKRLKFPRDLSLDP | Wildtype |
| ALMS1_wt | YSHTEKPGVFYQQVL | Wildtype |
| CDH12_wt | PVGTIIGAVTAQDLD | Wildtype |
| GLIS3_wt | IGKGSCNNLVVTSSP | Wildtype |
| HTR1E_wt | LDRYWAITNAIEYAR | Wildtype |
| MYT1L_wt | PIAAAEKLAKAQEKH | Wildtype |
| ONECUT2_wt | TLTPLQPLPPISTVS | Wildtype |
| WFS1_wt | SMVKLILVWLTAIVL | Wildtype |

Supplementary Table S9. D1313 neoepitopes (15mer, unless fitted to HLA molecules) derived from DNA exome sequencing derived. Red: marked point mutation as compared to the wt protein

| Name | Peptide | Mutation |
|---|---|---|
| TPR | TTNETATSDDGDEVF | c.6778G>A:p.Gly2260Ser |
| CCDC116 | EPCRSLYSNLPASRQ | c.1643C>G:p.Thr548Ser |
| SPEF2 | QRCFDIEMQYLNRRR | c.584A>T:p.Lys195Met |
| PTGIS | LHLEEMGMSEEMQAR | c.802G>A:p.Val268Met |
| STAB1 | FSFVRGQLLFKGCDV | c.79G>T:p.Val27Leu |
| C6 | CQCAPCPKNGRPTLS | c.1584T>A:p.Asn528Lys |
| B3GAT2 | VQKAELTGLANTFRQ | c.298C>G:p.Arg100Gly |
| SFXN5 | LGAVISADSIAVGLN | c.524T>A:p.Val175Asp |
| GOLGA6L10 | GGAEEAAGHSFRAAE | c.1387T>G:p.Cys463Gly |
| VLDLR | KSRLYWLASKLHMLS | c.1853A>C:p.Asp618Ala |
| C17orf53 | LLETCQNKLKPGSVL | c.1612G>A:p.Glu538Lys |
| FAM161B | AELFRKICIQMRALD | c.1138C>T:p.Arg380Cys |
| KIAA0368 | LQSQSWKVKAQGAIA | c.5143A>G:p.Met1715Val |
| THSD7B | QSCDPHTIQRRTRHL | c.3453G>A:p.Met1151Ile |
| PRKCE | PVLTLVDKAIVKQIN | c.2140G>A:p.Glu714Lys |
| PHEX | SYGAIGVTVGHEFTH | c.1730T>C:p.Ile577Thr |
| POM121L2 | LMLKPILWPLHNSEI | c.1615G>T:p.Gly539Trp |
| ZNF518B | DQLIKCPCRNQPVIV | c.2812C>T:p.Arg938Cys |
| HS3ST3A1 | LPQAIIIRVKKGGTR | c.475G>A:p.Gly159Arg |
| LRRN3 | TNNPRLSFIHPNAFF | c.971A>T:p.Tyr324Phe |
| TPR_wt | TTNETATGDDGDEVF | wildtype |
| CCDC116_wt | EPCRSLYTNLPASRQ | wildtype |
| SPEF2_wt | QRCFDIEKQYLNRRR | wildtype |
| PTGIS_wt | LHLEEMGVSEEMQAR | wildtype |
| STAB1_wt | FSFVRGQVLFKGCDV | wildtype |
| C6_wt | CQCAPCPNNGRPTLS | wildtype |
| B3GAT2_wt | VQKAELTRLANTFRQ | wildtype |
| SFXN5_wt | LGAVISAVSIAVGLN | wildtype |
| HLA-A*02:01_PKN2_2 | KLVISPLEL | P210L mutation peptide tailored for HLA-A*02:01 |
| HLA-A*02:01_POM121L2 | LMLKPILWPL | G539W mutation peptide tailored for HLA-A*02:01 |
| HLA-A*02:01_KIAA0368 | ALQSQSWKV | M715V mutation peptide tailored for HLA-A*02:01 |
| HLA-A*03:01_C17orf53 | RLLLETCQNK | E538K mutation peptide tailored for HLA-A*03:01 |
| HLA-B*07:02_C6 | CPKNGRPTL | N528K mutation peptide tailored for HLA-B*07:02 |
| HLA-B*07:02_HS3ST3A1 | RVKKGGTRAL | G159R mutation peptide tailored for HLA-B*07:02 |
| HLA-DRB1*11:01_SPEF2 | IEMQYLNRRRQNEI | K195M mutation peptide tailored for HLA-DRB1*11:01 |
| HLA-DQA1*05:01_TPR | SDDGDEVFVEA | G2260S mutation peptide tailored for HLA-DQA1*05:01 |

| | | |
|---|---|---|
| HLA-DQB1*03:01_PHEX | RSLSYGAIGVTVGHE | E714K mutation peptide tailored for HLA-DQB1*03:01 |
| HLA-DPA1*01:03_PKN2 | KLVISPLELRMEEL | P210L mutation peptide tailored for HLA-DPA1*01:03 |
| HLA-DPB1*04:02_ZNF518B | LKDPSIFQVARQLRLIAAKPDQLIKCPC | R938C mutation peptide tailored for HLA-DPB1*04:02 |
| HLA-DPB1*04:02_UQCRFS1 | FVSSMSASAVVLALAKIEIKLSD | D145V mutation peptide tailored for HLA-DPB1*04:02 |

Supplementary Table S10. D3731 neoepitopes (15mer, unless fitted to HLA molecules) derived from DNA exome sequencing. Red: marked point mutation as compared to the wt protein

| Name | Peptide | Mutation |
|---|---|---|
| AGAP10 | RRSNCTNRCDLGDTS | c.683A>G:p.His228Arg |
| AGK | GSRKVRNLKLHVEGT | c.1067C>T:p.Pro356Leu |
| C2orf81 | VLVSYPSAGGATRPS | c.932T>C:p.Val311Ala |
| RFC3 | TTPSKKKNEISTIAS | c.245T>A:p.Ile82Asn |
| KMT2A | TDSTQSAKSSPDEDT | c.7179C>G:p.Asn2393Lys |
| KRAS | KLVVVGARGVGKSAL | c.34G>C:p.Gly12Arg |
| TP53 | DDRNTFRRSVVVPYE | c.641A>G:p.His214Arg |
| AC016885.1 | ELPQHELWPEPPKQV | c.265C>T:p.Arg89Trp |
| ANKRD9 | ADGGPEAAGAARSRA | c.55T>G:p.Ser19Ala |
| COL4A1 | CLEEFRSVPFIECHG | c.4832C>T:p.Ala1611Val |
| DDX42 | PSAGAQGINNTASGN | c.2359G>A:p.Val787Ile |
| GCC1 | DEKNQPDSRLQELQE | c.952C>T:p.Pro318Ser |
| GOLGA6L10 | HEQEERLREQEERLC | c.772T>C:p.Cys258Arg |
| LMTK3 | VSSEYYICLEEHGSP | c.1708C>T:p.Arg570Cys |
| MYEF2 | GGHVPDMESGLMNLP | c.605G>A:p.Gly202Glu |
| PCDHGA4 | TQLFALNLRSGTLVT | c.236C>T:p.Pro79Leu |
| PFAS | ALPPGLSMHQALERV | c.1981G>A:p.Val661Met |
| PIWIL3 | LSRPLKEQRVEWLST | c.569G>A:p.Arg190Gln |
| PSD | QSWITRISVVAAMFS | c.2594A>G:p.Asn865Ser |
| RPS6KA2 | IDWNTLYWKEIKPPF | c.1006C>T:p.Arg336Trp |
| SLC39A5 | GTDITWMALLGDGLH | c.1160T>C:p.Val387Ala |
| SLC6A1 | LKGSLKQCIQVMVQP | c.1696C>T:p.Arg566Cys |
| SPRR2D | QPPPVCPKPKCPEPC | c.59C>A:p.Thr20Lys |
| TEX14 | QTALFVAVLLGLRKF | c.191C>T:p.Ala64Val |

| | | |
|---|---|---|
| TPO | WGKPEESHVSSVLEE | c.113G>A:p.Arg38His |
| TTC5 | HHVMDSVQQAKLAVQ | c.497G>A:p.Arg166Gln |
| HLA-DPA1*01:03_ANKRD9_1 | PEAAGAARSRAQKQCRKSSFAFYQAVRDLL | S19A mutation peptide tailored for HLA-DPA1*01:03 |
| HLA-C*08:02_ANKRD9_2 | GADGGPEAA | S19A mutation peptide tailored for HLA-C*08:02 |
| HLA-A*03:01_Mesothelin-GPI_1 | ILRPRFRREVEK | wildtype peptide tailored for HLA-A*03:01 |
| HLA-A*02:01_Mesothelin-GPI_11 | TLTAFYPGYL | wildtype peptide tailored for HLA-A*02:01 |
| HLA-A*02:01_Mesothelin-GPI_12 | SLSPEELSSV | wildtype peptide tailored for HLA-A*02:01 |
| HLA-DRB1*01:01_CD47 | LGLVYMKFVASNQKTIQ | wildtype peptide tailored for HLA-DRB1*01:01 |
| HLA-DQA1*01:01_Claudin18.2 | LWRSCVRESSGFTECRGYFTLLGLPAMLQA | wildtype peptide tailored for HLA-DQA1*01:01 |
| HLA-DQA1*01:01_EGFR | RGNMYYENSYALAVLSN | wildtype peptide tailored for HLA-DQA1*01:01 |
| HLA-DRB3*03:01_EpCam | FITSILYENNVITI | wildtype peptide tailored for HLA-DRB3*03:01 |
| HLA-DQA1*01:01_ERBB2 | LRENTSPKANKEILDEAYVMAGVGSPYV | wildtype peptide tailored for HLA-DQA1*01:01 |
| HLA-DRB3*03:01_FOLR1 | GAACQPFHFYFPTPTVLCNEIWTHSY | wildtype peptide tailored for HLA-DRB3*03:01 |
| HLA-DRB3*03:01_GPC3 | LNMEQLLQSASMELKF | wildtype peptide tailored for HLA-DRB3*03:01 |
| HLA-DRB1*01:02_PSMA | RGGMVFELANSIVLPF | wildtype peptide tailored for HLA-DRB1*01:02 |
| HLA-DQA1*01:01_Kras_17AA-4 | YKLVVVGARGVGKSA | G12R mutation peptide tailored for HLA-DQA1*01:01 |

Supplementary Table S11. D1688 neoepitopes (15mer, unless fitted to HLA molecules) derived from DNA exome sequencing. Red: marked point mutation as compared to the wt protein

| Name | Peptide | Mutation |
|---|---|---|
| CD1D | TVRSLKPSSQGTFSD | c.212G>C:p.Trp71Ser |
| TGFBR2 | LANSGQVRTARYMAP | c.1333G>A:p.Gly445Arg |
| ATP1A2 | PTEKLCFVGAHVYD | c.1752dup:p.Leu585AlafsTer6 |
| MATR3_1 | SAAGIGLLCCCYPVF | c.87dup:p.Ala30CysfsTer25 |
| MATR3_2 | KYASISWKDEPGYCT | c.87dup:p.Ala30CysfsTer25 |
| MUC6_1 | ATSFGLEPRGPAAPH | c.1519dup:p.Leu507ProfsTer37 |
| MUC6_2 | LPGLCHCWAPVQRSD | c.1519dup:p.Leu507ProfsTer37 |
| MUC6_3 | QRALRQLQRGHNG | c.1519dup:p.Leu507ProfsTer37 |
| RAB13 | AEDNFNNTSPPSELI | c.108_111del:p.Tyr37SerfsTer15 |
| SLC25A48_1 | GFFKGMSFPPRQHCR | c.196dup:p.Leu66ProfsTer17 |
| SLC25A48_2 | LQLRGVWGLQ | c.196dup:p.Leu66ProfsTer17 |
| ZNF678_1 | KVFNWWSQLTIRKFI | c.507_510del:p.Asn170IlefsTer26 |
| ZNF678_2 | LERNPTNVMNVTKFL | c.507_510del:p.Asn170IlefsTer26 |
| SACS | FVLNEKVAEFSGVLR | c.6257A>C:p.Asp2086Ala |
| COL19A1 | GSPGAPGLQGPPGPS | c.3338C>T:p.Pro1113Leu |
| EMILIN3 | LVGPGDRDRGPLTPP | c.719C>A:p.Ala240Asp |
| UNC13C | SQSTANELSTTLDSD | c.2531C>T:p.Ser844Leu |
| CHI3L2 | GWQDRGPNSYYNVEY | c.719G>A:p.Ser240Asn |
| ZNF644 | QKKTFMKESVVGSSK | c.1722C>A:p.Asp574Glu |
| WDFY4 | SLRTKQGSVVDVQKG | c.2380C>T:p.Pro794Ser |
| C10orf71 | SDPLLELLAEDLRTL | c.3389C>T:p.Ser1130Leu |
| HLA-B*44:02_MYH4 | HEATAAALW | R1197W mutation peptide tailored for HLA-B*44:02 |
| HLA-A*02:01_C10orf71 | LLAEDLRTL | S1130L mutation peptide tailored for HLA-A*02:01 |
| HLA-A*02:01_GDPD2 | SQSAGITGV | L468V mutation peptide tailored for HLA-A*02:01 |
| HLA-A*02:01_OR5I1 | SSVEIIFFI | C212F mutation peptide tailored for HLA-A*02:01 |
| HLA-B*44:02_ZNF585A | GERPCQSCPGEKLW | R38C mutation peptide tailored for HLA-B*44:02 |

| | | |
|---|---|---|
| HLA-DQB1*06:03_MYH4 | RRDLEESTLQHEATAAALWKKHAD | R1197R mutation peptide tailored for HLA-DQB1*06:03 |
| HLA-DRB1*13:01_CHI3L2 | SFDFHGSWEKPLITGHNSPLSKGWQDRGPN | S240N mutation peptide tailored for HLA-DRB1*13:01 |
| HLA-DPB1*16:01_ZNF644 | VKCPMVTSDIAQRKTQKKTFMKESVVGS | D574E mutation peptide tailored for HLA-DPB1*16:01 |
| HLA-DQA1*01:02_COL19A1 | PGLPGTSALGLPGSPGAPGL | P1113L mutation peptide tailored for HLA-DQA1*01:02 |

Supplementary Table S12. Top 10 CDR3 sequences for the TCR VA and VB chain in TIL expanded with the DPB1*04:02_UQCRFS1 peptide (FVSSMSASAVVLALAKIEIKLSD) from patient D1313

| **CDR3** | **TRAV** | **TRAJ** | **Proportion** |
|---|---|---|---|
| CAVGAIDNAGKSTF | TRAV8-3*02 | TRAJ27*01 | 16.67% |
| CAGVGGGTSYGKLTF | TRAV27*03 | TRAJ52*01 | 12.50% |
| CVVSANTGNQFYF | TRAV8-2*01 | TRAJ49*01 | 12.50% |
| CAVEDRSRGQNFVF | TRAV2*01 | TRAJ26*01 | 8.33% |
| CAASKGYSNYQLIW | TRAV13-1*02 | TRAJ33*01 | 8.33% |
| CAALCNFNKFYF | TRAV21*01 | TRAJ21*01 | 4.17% |
| CAVRDMRLAEGGADGLTF | TRAV3*01 | TRAJ45*01 | 4.17% |
| CAARGSGTYKYIF | TRAV29/DV5*01 | TRAJ40*01 | 4.17% |
| CVVHQAGGKLIF | TRAV12-1*01 | TRAJ23*01 | 4.17% |
| CAVRDMRLVGGGADGLTF | TRAV3*01 | TRAJ45*01 | 4.17% |
| **CDR3** | **TRBV** | **TRBJ** | **Proportion** |
| CASSWDRGIDYTF | TRBV6-3*01 | TRBJ1-2*01 | 15.00% |
| CASSPTGMNTEAFF | TRBV6-6*01 | TRBJ1-1*01 | 15.00% |
| CASSLSTSGSSYNEQFF | TRBV28*01 | TRBJ2-1*01 | 15.00% |
| CASSVGARDTQYF | TRBV9*01 | TRBJ2-3*01 | 15.00% |
| CAITAREGGTQYF | TRBV10-3*01 | TRBJ2-5*01 | 10.00% |
| CATTAREGETQYF | TRBV10-3*01 | TRBJ2-5*01 | 5.00% |
| CASSSGISYEQYF | TRBV5-4*01 | TRBJ2-7*01 | 5.00% |
| CSARDHVVPHF | TRBV20-1*01 | TRBJ1-4*01 | 5.00% |
| CSARPRGEMQPQHF | TRBV20-1*01 | TRBJ1-5*01 | 5.00% |
| CASSGMDYEQYF | TRBV2*01 | TRBJ2-7*01 | 5.00% |

Supplementary Table S13. CDR3 sequences for the TCR VA and VB chains in TIL expanded with Mesothelin_Pos106-120_WT (HRLSEPPEDLDALPL) peptide from D1313

| **CDR3** | **TRAV** | **TRAJ** | **Proportion** |
|---|---|---|---|
| CAYRGKAAGNKLTF | TRAV38-2/DV8*01 | TRAJ17*01 | 78.57% |
| CVVSPIGGSNYKLTF | TRAV10*01 | TRAJ53*01 | 7.14% |
| CASISSGSARQLTF | TRAV3*01 | TRAJ22*01 | 7.14% |
| CAVRDSNYQLIW | TRAV1-2*01 | TRAJ33*01 | 7.14% |
| **CDR3** | **TRBV** | **TRBJ** | **Proportion** |
| CASSYEGTGGYEQYF | TRBV6-5*01 | TRBJ2-7*01 | 33.33% |
| CASRQRQGAGEKLFF | TRBV6-6*01 | TRBJ1-4*01 | 16.67% |
| CASSVSFNLGGVTSEGYNEQFF | TRBV9*01 | TRBJ2-1*01 | 16.67% |
| CASSFSGFEQFF | TRBV12-4*01 | TRBJ2-1*01 | 16.67% |
| CASSLSTSGSSYNEQFF | TRBV28*01 | TRBJ2-1*01 | 16.67% |

Supplementary Table S14. Top 10 CDR3 sequences for the TCR VA and VB chain in TIL expanded with the LRP1B peptide (VSKRLKFSRDLSLDP) from Patient D1309

| **CDR3** | **TRAV** | **TRAJ** | **Proportion** |
|---|---|---|---|
| CATGTGTASKLTF | TRAV17*01 | TRAJ44*01 | 13.86% |
| CAMREVGDYKLSF | TRAV14/DV4*02 | TRAJ20*01 | 9.59% |
| CAMREVGDYKLSF | TRAV14/DV4*01 | TRAJ20*01 | 7.78% |
| CIVSLGGSQGNLIF | TRAV26-1*02 | TRAJ42*01 | 7.05% |
| CAASSTGGSQGNLIF | TRAV23/DV6*02 | TRAJ42*01 | 5.50% |
| CAARENTGNQFYF | TRAV23/DV6*02 | TRAJ49*01 | 4.35% |
| CAVEDQGYQKVTF | TRAV2*01 | TRAJ13*02 | 2.35% |
| CAARSYTDKLIF | TRAV23/DV6*02 | TRAJ34*01 | 1.95% |
| CALGPGSYQLTF | TRAV19*01 | TRAJ28*01 | 1.74% |
| CALSATNNNDMRF | TRAV19*01 | TRAJ43*01 | 1.64% |
| **CDR3** | **TRBV** | **TRBJ** | **Proportion** |
| CASSRTGIDSNQPQHF | TRBV6-1*01 | TRBJ1-5*01 | 14.44% |
| CSATTGDFSQETQYF | TRBV20-1*01 | TRBJ2-5*01 | 8.91% |
| CASSVGLAEEGDEQYF | TRBV10-2*01 | TRBJ2-7*01 | 6.38% |
| CASSPLSVNTEAFF | TRBV12-4*01 | TRBJ1-1*01 | 4.94% |
| CASSWTSGAAEQYF | TRBV5-1*01 | TRBJ2-7*01 | 3.64% |
| CASSYAGRRTSPLHF | TRBV6-5*01 | TRBJ1-6*01 | 2.73% |
| CASSLYGREDSYNEQFF | TRBV7-8*02 | TRBJ2-1*01 | 2.47% |
| CASSSELFQETQYF | TRBV6-5*01 | TRBJ2-5*01 | 2.02% |
| CASKEQGEPYEQYF | TRBV5-1*01 | TRBJ2-7*01 | 1.95% |
| CASSGGTSGQETQYF | TRBV9*01 | TRBJ2-5*01 | 1.82% |
